# Assessment of the Anti-Inflammatory, Antibacterial and Anti-Aging Properties and Possible Use on the Skin of Hydrogels Containing *Epilobium angustifolium* L. Extracts

**DOI:** 10.3389/fphar.2022.896706

**Published:** 2022-07-01

**Authors:** Anna Nowak, Martyna Zagórska-Dziok, Magdalena Perużyńska, Krystyna Cybulska, Edyta Kucharska, Paula Ossowicz-Rupniewska, Katarzyna Piotrowska, Wiktoria Duchnik, Łukasz Kucharski, Tadeusz Sulikowski, Marek Droździk, Adam Klimowicz

**Affiliations:** ^1^ Department of Cosmetic and Pharmaceutical Chemistry, Pomeranian Medical University in Szczecin, Szczecin, Poland; ^2^ Department of Technology of Cosmetic and Pharmaceutical Products, Medical College, University of Information Technology and Management in Rzeszow, Rzeszów, Poland; ^3^ Department of Experimental and Clinical Pharmacology, Pomeranian Medical University in Szczecin, Szczecin, Poland; ^4^ Department of Microbiology and Environmental Chemistry, Faculty of Environmental Management and Agriculture, West Pomeranian University of Technology, Szczecin, Poland; ^5^ Department of Chemical Organic Technology and Polymeric Materials, Faculty of Chemical Technology and Engineering, West Pomeranian University of Technology, Szczecin, Poland; ^6^ Department of Physiology, Pomeranian Medical University in Szczecin, Szczecin, Poland; ^7^ Department of Pharmaceutical Chemistry, Pomeranian Medical University, Szczecin, Poland; ^8^ Clinic of General Surgery, Minimally Invasive and Gastrointestinal, Pomeranian Medical University in Szczecin, Szczecin, Poland

**Keywords:** *E. angustifolium*, hydrogels, antioxidant, skin penetration, anti-aging, wound healing, anti-inflammatory

## Abstract

*Epilobium angustifolium* L. is an ethnomedicinal plant known as a medicinal plant in many regions of the world, among others, in various skin diseases. Despite the great interest in this plant, there are still few reports of biological activity of ready-made dermatological or cosmetical preparations containing the *E. angustifolium* extracts. The antioxidant, anti-ageing, anti-inflammatory, antibacterial properties and toxicity, wound healing, and skin permeation of topical hydrogels containing *E. angustifolium* extracts (HEas) was assessed. First, the plant extracts were prepared using three solvents: 70% (v/v) ethanol, 70% (v/v) isopropanol and water, next by preparing hydrogels witch by dry extracts (HEa-EtOH), (HEa-iPrOH) and (HEa-WA), respectively. Finally, the content of selected phenolic acids in the HEas was evaluated by high-performance liquid chromatography (HPLC). All the HEas were characterized by high antioxidant activity. The most increased antibacterial activity was observed for a strain of *Streptococcus pneumoniae* ATCC 49619, *Escherichia coli*, *Enterococcus faecalis* ATCC 29212, *Enterococcus faecium*, *Sarcina lutea* ATCC 9341 and *Bacillus pseudomycoides,* while the strains of *Streptococcus epidermidis*, *Bacillus subtilis*, and *Staphylococcus aureus* were the least sensitive. All the HEas showed a reduction in the activity of lipoxygenase enzymes, proteases, and inhibition of protein denaturation. The HEa-EtOH and HEa-iPrOH also enhanced the wound healing activity of HDF cells. Additionally, *in vitro* penetration studies were performed using the Franz diffusion cells. These studies showed that the active ingredients contained in *E. angustifolium* penetrate through human skin and accumulate in it. Furthermore, the hydrogels containing *E. angustifolium* extracts showed a broad spectrum of activity. Therefore, they can be considered as an interesting alternative for dermatologic and cosmetic preparations.

## 1 Introduction

In recent years, a growing interest in the search for practical and safe dermatological preparations containing active ingredients with multiple effects was observed (Zagórska-Dziok et al., 2021a). Therefore, more and more attention is paid to the use of natural substances, including plant extracts. Due to the abundance of secondary metabolites they contain, plant extracts can play simultaneously multiple roles, such as antioxidant, antibacterial, anti-inflammatory, and anti-ageing (Fibrich et al., 2020; Nowak et al., 2021d). Recently, hydrogels have become more and more popular because they are often characterized by a simple composition, high water content and high biocompatibility with skin cells. Many authors have assessed the biological activity of hydrogels containing plant extracts. For example, the hydrogels films composed of agarose, κ-carrageenan and glycerol containing the aqueous extract from *Cryphaea heteromalla* showed solid antioxidant properties (Ditta et al., 2020). A carbomer-based hydrogel containing 2% of *Punica granatum* peels ethanolic extract completely healed the chronic leg ulcer in a 76-year-old woman (Fleck et al., 2016). The hydrogels containing 5% and 10% hexane extracts from *Moringa oleifera* seeds showed significant healing activity for excised and incised wounds in albino mice (Ali et al., 2020).

*Epilobium angustifolium* L. (Onagraceae) is an ethnomedicinal plant known and used in many world regions. This plant occurs mainly in North America, Asia and Europe. It is well known as a natural anti-inflammatory (Ruszová et al., 2014; Zagórska-Dziok et al., 2021b; Nowak et al., 2021d), antioxidant (Dacrema et al., 2020; Lasinskas et al., 2020; Szwajgier et al., 2021), antibacterial (Ferrante et al., 2020; Nowak et al., 2021a), analgesic (Tita et al., 2001) and anti-cancer drug (Kadam et al., 2018; Adamczak et al., 2019). I folk medicine, this plant has been used to treat eczema, acne, minor burns, skin rashes, and ulcers. For example, the North American Indians used *Epilobium* species to heal infected wounds, where they macerated the root and then applied it to boils and infections. In contrast, the leaves were used topically for bruises. The root has also been used as an antiseptic to treat open wounds infection (Roges, 2014; Mohammadi Bazargani et al., 2021). An ointment made from the leaves was used for skin diseases in children (Karakaya et al., 2020). Despite many studies on the therapeutic properties of this plant, there are still not many reports on the biological activity of ready-made dermatological or cosmetic preparations containing *E. angustifolium*. Our previous studies showed that adding ethanol extract from *E. angustifolium* to bacterial cellulose membranes enriches them with antioxidant activity (Nowak et al., 2021c). In addition, we have shown that the valuable phenolic acids in this plant’s extract penetrate the skin and accumulate in it (Nowak et al., 2021c; 2021a; 2021d). However, in earlier studies, only ethanol extract was used. Whereas, it is known that the correct selection of the solvent for the preparation of the plant extract is significant and may essentially decide about the biological activity of the ready-made preparations applied to the skin (Karakaya et al., 2020).

Despite the fact that *E. angustifolium* is a plant that has been used for a very long time, in literature there are not many reports on the comprehensive use of this plant in skin care and treatment, and the results of the conducted research are usually limited to assessing only of selected parameters. The conducted research most often concernsed the evaluation of alcoholic or water extracts from this plant, which confirmed its the antioxidant, anti-inflammatory, antibacterial and anti-aging effects (Ruszová et al., 2014; Karakaya et al., 2020; Nowak et al., 2021a), which in the context of the skin application is very important. Whereas, the number of research with the participation of readymade preparations is small. In the case in the case of dermocosmetics containing plant extracts, the carrier used can significantly affect the release and penetration of active substances through the skin. The plant extract incorporated into the substrate is often subjected to many technological processes, such as evaporation, which may result in the loss of some secondary metabolites. Therefore, the aim of our work was to estimate the biological activity of ready-made hydrogels containing EA extracts prepared with the use of three different solvents (70% ethanol, 70% isopropanol, and water). We assessed them of biological activity covering antioxidant, antibacterial, anti-inflammatory, anti-ageing properties, and cytotoxicity to human fibroblasts. We also estimated the permeation of selected phenolic acids through the pigskin and their accumulation in it.

## 2 Materials and Methods

### 2.1 Chemicals

2,2-diphenyl-1-picrylhydrazyl (DPPH), 6-hydroxy-2,5,7,8-tetramethylchroman-2-carboxylic acid (Trolox), 2,20-azino-bis(3-ethylbenzothiazoline-6-sulfonic acid) (ABTS), hydroxyethyl cellulose (HEC), perchloric acid, 3,4-dihydroxybenzoic acid, casein, potassium persulfate, sodium linoleic salt, bovine serum, Neu reagent, diclofenac and Tris-HCl buffer were purchased from Sigma Aldrich (Steinheim am Albuch, Germany), Folin–Ciocalteu reagent, gallic acid, 4-hydroxybenzoic acid, 3-hydroxybenzoic acid, disodium phosphate, propylene glycol, and potassium dihydrogen phosphate from Merck (Darmstadt, Germany), acetic acid, aluminium chloride, hydrochloric acid, sodium sulphate anhydrous, sodium lauryl sulfate, phosphate-buffered saline (PBS) as well as propylene glycol, ethanol, methanol and isopropanol were from Chempur (Piekary Śląskie, Poland), whereas acetonitrile for HPLC from J.T. Baker, (the Netherlands). All reagents were of analytical grade.

### 2.2 Plant Material and Extraction

The plant material was the herb, including the stem, leaves, and flowers of *E. angustifolium*. The plant materials were collected from the natural state during the flowering phase in July in Poland in 2020 (N 53°23′18″, E 14°28′56″). The plants were selected randomly from different, near-located places. Six samples were harvested and combined into one collective sample. The plant material was identified by PhD Anna Nowak, who graduated from Agriculture University, Szczecin, Poland. The plant material was dried at room temperature in a well-ventilated area to a constant weight. Samples were deposited in the plant mate-rial storage room (Vouchr No. EAE-AM2020-03) at the Chair and Department of Cosmetic and Pharmaceutical Chemistry of the Pomeranian Medical University. The plant material was ground in the grinder and sieved using a circular-hole screen (8 mm mesh). Next, three types of extracts were prepared. Five grams of dried raw material were extracted with 100 ml 70% (v/v) ethanol, 100 ml 70% (v/v) isopropanol, and 100 ml water for 60 min in an ultrasonic bath at a frequency of 40 kHz. Then, obtained extracts were collected and filtered three times through Whatman filter paper No. 10. The extraction efficiency for the ethanol extract was 85.4%, for the isopropanol extract it was 87.1%, and for the water extract was 94.5%. The extracts were evaporated under reduced pressure at 40°C. The samples were stored in the dark at 4°C until the preparation of hydrogels.

### 2.3 Prepared Hydrogel

**FIGURE 1:**
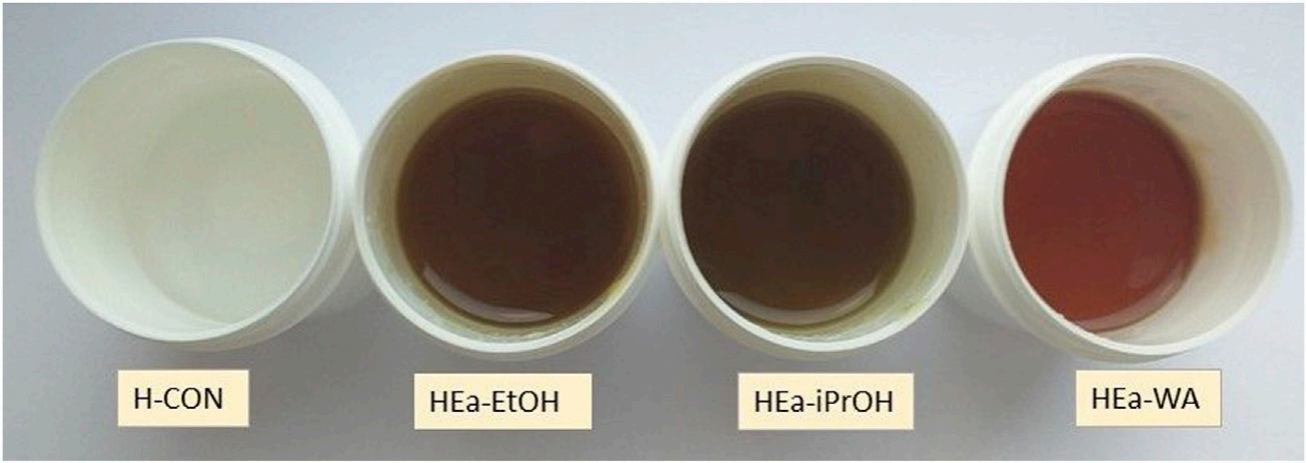
The hydrogels used in the study. HEa-EtOH—hydrogel containing dry ethanolic extract of *E. angustifolium*; HEa-iPrOH, hydrogel containing dry isopropanol extract of *E. angustifolium*; HEa-WA, hydrogel containing dry water extract of *E. angustifolium*; H–CON, hydrogel without *E. angustifolium* extract.

The hydrogels were prepared according to a modified procedure by Zagórska-Dziok et al. (Zagórska-Dziok et al., 2021a). Sequentially, an aqueous solution of hydroxyethyl cellulose (HEC) was prepared. The HEC was added to water and mixed on a mechanical stirrer (Chemland, Stargard, Poland) using a stirrer and stirring speed of 250 rpm. Then, the polymer solution was heated to 60°C and cooled to room temperature while constantly stirring. Dry extracts of *E. angustifolium* were added to the hydrogels by dissolving them in a propylene glycol solution (Fibrich et al., 2020). The dissolved dry plant extracts were added after all hydrogels cooled down. Three hydrogels were obtained, containing dry ethanol extract (HEa-EtOH), dry isopropanol extract (HEa-iPrOH) and dry water extract (HEa-WA). The hydrogel without plant extract was also prepared (H-CON)—Figure 1. The compositions of the hydrogels are shown in Table 1.

**TABLE 1:** The *E. angustifolium* hydrogels composition.

Ingredient		HEa-EtOH	HEa-iPrOH	HEa-WA	H-CON
*E. angustifolium*	Dry ethanol extract^a^	5	−	−	−
	Dry isopropanol extract^a^	−	5	−	−
	Dry water extract^a^	−	−	5	−
Glycol propylene^a^	20	20	20	20	
Hydroxyethylcellulose^a^	2	2	2	2	
Water	ad 100	ad 100	ad 100	ad 100	

a The amount of components are expressed in g; HEa-EtOH—hydrogel containing dry ethanolic extract of *E. angustifolium*; HEa-IsoPr—hydrogel containing dry isopropanol extract of *E. angustifolium*; HEa-WA—hydrogel containing dry water extract of *E. angustifolium*, H–CON, hydrogel without *E. angustifolium* extract.

### 2.4 Identification of Phenolic Acids in Hydrogels

The hydrogel preparation for HPLC analysis was done according to the modified method of (Fibrich et al., 2020). Namely, the concentration of phenolic acids in all hydrogels was determined by sampling 10 µl of the hydrogel from three different locations in the container. These samples were diluted 50 times in PBS, then centrifuged until the hydrogel dissolved and injected for HPLC analysis. The concentration of phenolic acids was determined by high-performance liquid chromatography (HPLC), using the HPLC system from Knauer (Berlin, Germany). The tested components were separated on a 125 mm × 4 mm column containing Hyperisil ODS, particle size 5 µm. The mobile phase consisted of acetonitrile, 1% acetic acid, and MeOH (45:45:10 by vol.), and the flow rate was 1 ml/min. Twenty microliters (µl) of the sample were injected into the column. The correlation coefficient of the calibration curve was 0.9964 for gallic acid (GA) (y = 136699x + 1.2732, Rt-4.050 min); 0.999 for 3,4-dihydroxybenzoic acid (3,4-DHB) (y = 40070x − 1.16783, Rt −4.850 min); 0.9998 for 3-hydroxybenzoic acid (3-HB) (y = 45422x + 3.7381, Rt −8.000 min); 0.999 for 4-hydroxybenzoic acid (3-HB) (y = 28271x + 1.3739, Rt −8.7683 min). All samples were analyzed three times.

### 2.5 Stability of Hydrogels

The stability of all hydrogels was tested to the modified method at Muthachan and Tewtrakul (Muthachan and Tewtrakul, 2019). The separation of the hydrogels was evaluated by centrifuge test. The hydrogel samples (3 g) were centrifuged (MPW-223e, Mechanika Precyzyjna, Warsaw, Poland) at 4,000 rpm at 25°C for 10 min to establish the possibility of preparation breaking. Moreover, the stability of all hydrogels was also evaluated using the heating-cooling test: incubation at 45°C (Drying Oven, DHG-9075A) for 48 h, followed by incubation at 4°C (in the refrigerator) for 48 h. The test was performed for six cycles. All hydrogels containing *E. angustifolium* extract kept at heating-cooling condition were confirmed by visual appearance.

### 2.6 Antioxidant Activity Determination of Hydrogels

The scavenging activity of DPPH (2,2-diphenylo-1-pikcrylhydrazyl) stable free radicals was measured according to the modified method described earlier (Nowak et al., 2021a; Muzykiewicz-Szymańska et al., 2021). The sample of 0.15 ml of the analyzed hydrogels was mixed with 2.85 ml of 0.3 mmol/L DPPH radical solution dissolved in 96% (v/v) ethanol. Measurement of absorbance at 517 nm against 96% (v/v) ethanol was performed after 10 min of incubation in the dark at room temperature using Hitachi UV-Vis Spectrophotometer U-5100. As a reference 6-hydroxy-2,5,7,8-tetramethylchroman-2-carboxylic acid (Trolox) was applied. The results are expressed in mg Trolox/g hydrogel.

ABTS (2,2′-azino-bis(3-ethylbenzothiazoline-6-sulfonic acid)) radical scavenging activity was evaluated as described previously (Nowak et al., 2021d; Muzykiewicz-Szymańska et al., 2021). The stock solution was a 7 mmol/L solution of ABTS in a 2.45 mmol/L aqueous solution of potassium persulfate. After dissolving the components, the solution was incubated for 24 h, in the dark at room temperature, then diluted with 50% (v/v) methanol to obtain a working solution. An aliquot of 2.5 ml of working ABTS solution and 0.025 ml of analyzed hydrogels was introduced into the spectrophotometric cuvette. After 6 min incubation in the dark at room temperature absorbance at 734 nm was measured. The Trolox was used as a standard, and the results were expressed in mg Trolox/g hydrogel.

Total polyphenol content was determined with the Folin–Ciocalteu method as described previously (Nowak et al., 2019). Shortly, to 0.15 ml of the hydrogels sample, 0.15 ml of tenfold diluted Folin–Ciocalteu reagent, 1.35 ml of 0.01 M sodium carbonate solution, and 1.35 ml of water were added and mixed thoroughly and was then incubated for 15 min at room temperature. After this time, the spectrophotometric measurement was carried out at 765 nm. Gallic acid was applied as a standard, and results were expressed as gallic acid equivalents (GA) in mg GA/g hydrogel.

For analyses of antioxidant activity, three independent measurements were made.

### 2.7 Anti-Inflammatory Properties

#### 2.7.1 Inhibition of Proteinase Activity

The evaluation of the possibility of proteinase inhibition by the analyzed hydrogels was carried out according to the method described by Sakat (2010), with minor modifications by Gunathilake et al. (2018). In the first step, the reaction solution was prepared by mixing a 1% trypsin solution dissolved in 20 mM Tris-HCl buffer (pH 7.4) and hydrogel test samples (at concentrations of 0.1, 1.0, and 5.0%). The solution was then incubated at 37°C for 5 min. In the next step, 0.8% (w/v) casein will be added, and the resulting mixture is incubated for another 20 min. After this time, 70% perchloric acid was added to stop the enzyme reaction. The obtained mixture was centrifuged and the absorbance of the samples was measured at *λ* = 210 nm using a DR600 UV-Vis spectrophotometer (Hach Lange, Wrocław, Poland). The reaction buffer was used as a blank. A phosphate buffer solution was used as a control. Three independent experiments were performed in which all samples were tested in triplicate. The percentage inhibition of protein denaturation by the analyzed hydrogels was calculated according to the following equation (Eq. 1):

$$\text{\% }inhibition\;of\;proteinase\;activity\;=100\text{ x }\frac{1-\text{A}2}{\text{A}1}$$ (1)

where: A_1_ is the absorbance of the control sample; A_2_ is the absorbance of the test sample.

#### 2.7.2 Inhibition of Lipoxygenase Activity

The possibility of inhibiting the lipoxygenase enzyme was assessed based on the method described by Ziemlewska et al. (2021). In the first step, on a standard 96-well plate, 10 µl of each concentration of test hydrogels (0.1, 1.0, and 5.0%) were mixed with 160 µl of 100 mM PBS and 20 µl of soybean lipoxygenase solution (167 U/ml). The prepared plates were incubated at 25°C for 10 min. Then 10 µl of sodium linoleic salt was added to the wells to initiate the enzymatic reaction. The absorbance of the samples was then measured every minute for 3 min. Measurements were made at *λ* = 234 nm using a microplate reader (Thermo Fisher Scientific, Waltham, MA, United States). Diclofenac at a 500 μg/ml concentration was used as a control sample. As part of the work, three independent experiments were performed in which all samples were tested in triplicate. The percentage of inhibition of lipoxygenase activity by the analyzed hydrogels was calculated from Eq.2:

$$\text{\% }inhibition\;of\;lipoxygenase\;activity=\frac{\text{Ac}-\text{As}}{\text{Ac}}\text{ x }100\text{\%}$$ (2)

where: As is the absorbance of the tested sample; Ac is the absorbance of the control sample.

#### 2.7.3 Assessment of Inhibition of Protein Denaturation

To assess the anti-inflammatory properties of the tested hydrogels with three types of *E. angustifolium* extracts, the methodology described by Sarveswaran et al. was used Sarveswaran (2017). As part of this assay, the possibility of inhibiting the denaturation of bovine serum albumin (BSA) by the tested hydrogels was assessed. In the first step, 1000 µl of test hydrogels (H-CON, HEa-WA, HEa-EtOH, and HEa-iPrOH) at concentrations of 0.1, 1.0, and 5.0% were mixed with 450 µl of 5% aqueous BSA solution and 1400 µl of phosphate-buffered saline (PBS, pH 6.4). The mixtures were incubated at 37°C for 15 min. The samples were then heated to 70°C for 5 min, and then the reaction solutions were cooled in an ice bath to 25°C. Then, the absorbance of the prepared samples was measured at λ = 660 nm using the DR600 UV-Vis spectrophotometer (Hach Lange, Wrocław, Poland). The positive control was acetylsalicylic acid (aspirin) at a 500 μg/ml concentration. As part of the analysis, three independent experiments were performed in which each sample was tested in three replications. The ability to inhibit protein denaturation by tested hydrogels was calculated based on Eq. 3:

$$\text{\% inhibition of }denaturation=\frac{1-\text{As}}{\text{Ac}}\text{ x }100\text{\%}$$ (3)

where: As is the absorbance of the tested sample; Ac is the absorbance of the control sample.

### 2.8 Biocompatibility Study

The biocompatibility study was performed on primary human dermal fibroblast (HDF) cells derived from the skin according to the protocol approved by the Ethical Committee of Pomeranian Medical University in Szczecin (KB-0012/02/18). The detailed protocol of fibroblasts isolation, authentication and cell culture conditions are provided in Supplementary Materials. The cell viability was evaluated using a ready-to-use resazurin-based PrestoBlue™ HS Cell Viability Reagent (Thermo Fisher Scientific, United States). Viable cells continuously convert resazurin to highly fluorescent resorufin, which is directly correlated to the number of metabolically active cells. Human dermal fibroblasts were seeded in 96-well black microplates (Greiner, Austria) at a density of 3 × 10^3^ cells/well and allowed to adhere for 24 h. Afterwards, the cell culture medium was removed and replaced with 100 µl of the fresh medium containing 0.01, 0.1, 0.2, 0.5, 1.0, 2.5, and 5.0% of the tested hydrogels (HEa-WA, HEa-EtOH, and HEa-iPrOH) corresponding to 5, 50, 100, 250, 500, 1250, and 2,500 μg/ml of *E. angustifolium* extracts. The hydrogel solutions were prepared in the complete cell culture medium by capillary pistons (Gilson MICROMAN™, United States) and sterilized using membrane filters (0.22 µm). The cells with the medium were used as the negative control, and cells treated with hydrogel (5%) without HEas were used as vehicle control (H-CON). After 24 h of treatment, the hydrogel solutions/medium were replaced with fresh medium (90 µL), and PrestoBlue reagent (10 μl) was added to each well and incubated for 30 min. The fluorescence was measured using a spectrophotometric microplate reader (Infinite 200 Pro, Tecan, Switzerland) at ex/em: 560/594 nm. The results were normalized to the negative control (100% viability). The readings were acquired from at least three independent experiments (each conducted in triplicate).

In the same conditions (after 24 h of treatment but in clear cell culture microplates) the lactate dehydrogenase (LDH) leakage was determined using a commercially available kit CytoTox96 Non-Radioactive Cytotoxicity Assay (LDH, Promega, United States). The loss of intracellular LDH and its release into the culture medium indicate irreversible cell death due to cell membrane damage. According to the manufacturer’s protocol, 50 µl aliquots from all test and control wells were transferred into fresh 96-well flat clear bottom plates after treatment. Next, the reconstituted Substrate Mix (50 µl) was added to each well and incubated at room temperature for 30 min, covered with foil for light protection. Finally, 50 µl Stop Solution was added to each well, and the absorbance was measured at 490 nm using a spectrophotometric microplate reader (Infinite 200 Pro, Tecan, Switzerland). The untreated cells were used as the negative control and cells with 10 µl Lysis Solution as the positive control (maximum LDH release). The tested hydrogels in the medium (0.01–5.0%) without cells were blanks. The readings were acquired from three independent experiments (each conducted in triplicate). The percentage of cytotoxicity was calculated using the following Eq. 4:

$$\text{\% viability}=100-\frac{\text{experimental LDH release }–\text{ blank}}{\text{maximum LDH release }–\text{ blank}}\text{ x }100\text{\%}$$ (4)

Additionally, optical microscopy imaging of human dermal fibroblasts after 24 h of treatment was performed using Smart Fluorescent Cell Analyzer Micro-scope JuLi (Korea).

### 2.9 Wound Healing Assay

The wound healing activities of hydrogels containing 0.2% (100 μg/ml of EA extracts) were evaluated using scratch (cell migration) method according to a previously described protocol by Sudsai et al. (Muthachan and Tewtrakul, 2019; Srirod and Tewtrakul, 2019) with minor modifications. Human dermal fibroblasts were seeded in 24-well plates at a density of 3 × 10^4^ cells/well and cultured as above, for about 48 h. Afterwards, a scratch was made with a sterile 200 μl tip, and the cells were then washed twice with PBS (to remove detached cells and other cellular debris) and treated with 0.2% of HEa-WA, HEa-EtOH, and HEa-iPrOH (100 μg/ml of EA extracts) for 12 h at standard cell culture conditions. Images were acquired every 30 min using Smart Fluorescent Cell Analyzer Micro-scope JuLi (Korea) with time-lapse imaging options. In addition, a percentage of the closed area was analyzed by ImageJ software with ImageJ/Fiji® plugin (Suarez-Arnedo et al., 2020) and compared with the value obtained at the time 0. An increase in the percentage of the closed area indicated the cells migration.

### 2.10 Antimicrobial Analysis of Hydrogels

The antimicrobial activity of hydrogels was tested against 11 strains of bacteria. The following microbial strains were used: *Escherichia coli, Sarcina lutea* ATCC 9341*, Serratia marcescens, Enterococcus faecalis* ATCC 29212*, Enterococcus faecium, Streptococcus pneumoniae* ATCC 49619*, Pseudomonas fluorescens, Bacillus subtilis, Bacillus pseudomycoides, Staphylococcus aureus, and Streptococcus epidermidis.* The sensitivity of the test microorganisms to the tested substances was determined by the method of diffusion into the agar medium using the well variant (Valgas et al., 2007). TSA (Tryptone Soya Agar) medium was used for bacterial cultivation, while in the case of *E. coli*, TBX (Tryptone Bile X-glucuronide Agar) chromogenic medium. The appropriate medium (20 ml) was poured into Petri dishes with a diameter of 90 mm. After the medium had solidified, 4 wells with a diameter of 6 mm were cut out using a sterile plug. Next, 0.1 ml of 24-h bacterial culture in a liquid tryptone-soybean (TSB) medium with 0.25% Tween 20 was introduced into the prepared dishes. The inoculum was evenly spread over the surface of the medium. The density of the bacterial cultures ranged from 1−5 × 10^7^ CFU per mL. Plates of inoculated strains were allowed to absorb the fluid for about 30 min entirely, and then 10 µl of each hydrogel was introduced into each well. Incubation of bacterial cultures was carried out for 72 h at 30°C and *E. coli* bacteria at 37°C. The inhibitory effect of the test substances was assessed based on the zone of inhibition of the growth of the culture. Measurements were taken every 24 h, and the results after 72 h were used for final analyzes.

### 2.11 *In vitro* Skin Permeation Studies

In the *in vitro* permeation experiments, an abdomen porcine skin was used due to their similar permeability to human skin (Jacobi et al., 2007; Khiao In et al., 2019). The skin for the experiment was prepared and was stored as in the previous studies (Nowak et al., 2021c; Ossowicz-Rupniewska et al., 2021). The permeation experiments were performed in the Franz diffusion cells (SES GmbH Analyse Systeme, Bechenheim, Germany) with a diffusion area of 1 cm^2^. The acceptor chamber was filled with PBS solution (pH 7.4). In each diffusion unit, a constant temperature of 37.0 ± 0.5°C was maintained (Fibrich et al., 2020). The acceptor chamber content was stirred with a magnetic stirring bar at the same speed for all cells. The donor chamber volume was 2 ml, and the volume of the acceptor chamber was 8 ml. Undamaged pieces of skin were placed in the Franz diffusion cell between donor and acceptor chamber. The skin integrity was measured using an LCR meter 4,080 (Voltcraft LCR 4080, Conrad Electronic, and Germany), as in our previous study (Nowak et al., 2021d; Ossowicz-Rupniewska et al., 2021). Next, a defined dose (1 g) of each hydrogel was applied to the skin’s outer side in the donor compartment. The penetration study was carried out for 24 h. At the time points of 1, 2, 3, 5, 8, and 24 h, 0.3 ml of acceptor samples were withdrawn, and the chamber was refilled with the same volume of a fresh portion of PBS pH 7.4. The HPLC method was applied to evaluate the phenolic acid concentrations in the acceptor phase. The cumulative mass (µg) of each phenolic acid studied was calculated based on the obtained concentration. The following permeation parameters, such as fluxes of phenolic acids from HEas through the skin (J_SS_), the permeability coefficient (K_P_), the time required to reach steady-state permeation (lag time—L_T_), the diffusion coefficient (D), the skin partition coefficient (K_m_), and the percentage of the applied dose after 24 h (Q%24 h) were determined. J-shaped profiles and following Eqs 4, 5 were used to determine the permeation parameters:

$$\mathrm{A=}J_{\mathrm{SS}}\;\left(t-L_{T}\right)$$ (5)

where: A is the cumulative amount (in μg cm^−2^) of tested phenolic acids permeating into the receptor compartment; J_ss_ is the steady-state flux (in μg cm^−2^ h^−1^); t is the time (h), and L_T_ is the lag time (h).

The steady-state flux was estimated from the slope of the linear portion of the plot of cumulative mass in the acceptor phase over time. The lag time (L_T_) was determined from the *x*-intercept of the linear portion of the plot of cumulative mass in the acceptor phase over time and was used to calculate the diffusion coefficient (K_P_) as follows:

$$K_{p}=J_{\mathrm{SS}}/C$$ (6)

where: C is the concentration in the donor phase.

After 24 h of the experiment, each skin sample was removed. In order to remove hydrogel residues from the skin, they were few times rinsed with 0.5% sodium lauryl sulfate solution. The skin samples were divided into two lots. The first was submitted for microscopic analysis, and the other was tested for accumulation of phenolic acids.

### 2.12 Accumulation of the Phenolic Acids in the Skin

Assessment of accumulation of active compounds in the skin was carried out similarly to our previous studies (Nowak et al., 2021a). In brief, after penetration, the skin was cut around the diffusion area (1 cm^2^) and incubated in 2-ml methanol for 24 h. After this time, skin samples were homogenized using a homogenizer (IKA®T18 digital ULTRA TURRAX, Staufen, Germany). The supernatant was collected for the HPLC analyses. Accumulation of the phenolic acids in the skin was calculated by dividing the amount of the substances remaining in the skin by the mass of the skin sample and was expressed as the mass of phenolic acid per mass of the skin (µg/g).

### 2.13 Fluorescent Microscopy

The skin samples removed from the Franz diffusion cell were fixed in 4% buffered para-formaldehyde for 24 h. Further skin samples were dehydrated in alcohols from 50% to 99.9% and xylene and then were embedded in paraffin blocks. Paraffin blocks were cut on a rotary microtome to 5 μm thick sections and placed on histological slides. For polyphenol visualization in histological sections, slides were rehydrated, starting with xylene and alcohols (99.9%–70%) and finished with a deionized water wash. After deparaffinization, samples were mounted with a fluorescent mounting medium. The Neu reagent (2-aminoethyl diphenylborinate 1% in methanol) was used to differentiate the polyphenols (Martimestres et al., 2007). Sections were scanned with a confocal microscope (FV-1000 Olympus) with 405 nm diode laser, Olympus IX81 inverted microscope, and FV10-ASW 4.2 software (Olympus).

### 2.14 ATR-FTIR Studies

The analyses on the effect of the HEas on the skin was assessed using the total reflection-Fourier transform infrared spectroscopy (ATR-FTIR), similarly to that reported in our previous study (Nowak et al., 2021b). In brief, the pure skin was cut into 1 cm^2^ piece and applied to the HEas for 24 h. Then the skin samples were washed with isopropanol, blotted dry, and air-dried for 2 h. An ATR unit obtained the spectra using Thermo Scientific Nicolet 380 spectrometer (Thermo Fisher Scientific, Waltham, MA, United States). The recorded spectrum represented an average of 32 scans obtained with a 4 cm^−1^. The spectra were collected in the wavenumber range of 4,000–400 cm^−1^. The internal reflectance element (IRE) used in this study was an ATR diamond plate. The skin was carefully mounted on the IRE. For comparison, the analysis was also performed for clean skin, not treated with a hydrogel.

### 2.15 Statistical Analysis

Results are presented as the mean ± standard deviation (SD). In addition, a one-way analysis of variance (ANOVA) was used. Tukey’s test evaluated the significance of differences between individual groups (α = 0.05). Statistical calculations were done using Statistica 13 PL software (*StatSoft*, Krakow, Poland).

## 3 Results

### 3.1 Chemical Composition of Hydrogels

**FIGURE 2:**
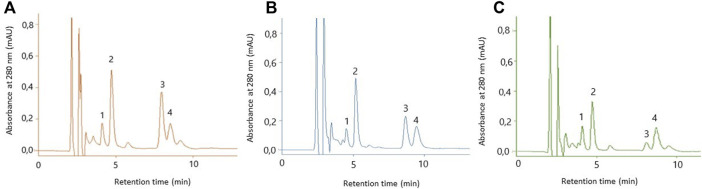
HPLC chromatogram of hydrogels. The hydrogel contained dry ethanol extract of *E. angustifolium* **(A)**; the hydrogel containing dry isopropanol extract of *E. angustifolium* **(B)**; The hydrogel contained dry water extract of *E. angustifolium* **(C)**, **1**—gallic acid; **2**–3,4 -hydroxybenzoic acid; **3**–3-hydroxybenzoic acid; **4**–4-hydroxybenzoic acid.

The content of selected phenolic acids in HEas is presented in Table 2 and Figure 2. The following phenolic acids were found: GA; 4-HB; 3-HB and 3,4-DHB. The 3,4-DHB was the most abundant, followed by 3-HB and 4-HB (Table 2).

**TABLE 2:** Content of selected phenolic acids in HEas.

Phenolic acid		HEa-EtOH	HEa-iPrOH	HEa-WA	H-CON
GA	mg/100 ml	3.37 ± 0.02^b^	2.76 ± 0.19^a^	2.385 ± 0.02^a^	nd
3,4 -DHB		21.97 ± 0.26^b^	20.46 ± 0.32^b^	9.69 ± 0.12^a^	nd
4-HB		8.10 ± 0.34^a^	8.84 ± 0.34^ab^	9.61 ± 0.34^a^	nd
3-HB		18.13 ± 0.33^b^	16.70 ± 0.33^b^	4.17 ± 0.02^a^	nd

GA, gallic acid; 3,4-DHB, 3,4-dihydroxybenzoic acid; 4-HB, 4-hydroxybenzoic acid; 3-HB, 3-hydroxybenzoic acid; HEa-EtOH, hydrogel containing dry ethanolic extract of *E. angustifolium*; HEa-iPrOH, hydrogel containing dry isopropanol extract of *E. angustifolium*; HEa-WA, hydrogel containing dry water extract of *E. angustifolium*; H–CON, hydrogel without *E. angustifolium* extract. Different letters indicate significant differences between the HEas, α = 0.05. Values are the mean of three replicate determinations (*n* = 3).

### 3.2 Stability Test of Hydrogels

All analyzed hydrogels showed good physical properties. No separation of the plant extract was observed after the vortex test. No change in the hydrogels’ colour and the odour was found compared to the hydrogels before to the heating-cooling test.

### 3.3 Biological Activity of Hydrogels

#### 3.3.1 Antioxidant Activity

The antioxidant activity and the total content of polyphenols in all analyzed hydrogels are presented in Table 3. All hydrogels containing plant extracts were characterized by antioxidant activity. The highest activity, measured by DPPH and ABTS methods, was observed for HEa-iPrOH > HEa-EtOH > HEa-WA. Similarly, the higher total polyphenol content was found for HEa-iPrOH and HEa-EtOH than in HEa-WA (Table 3).

**TABLE 3:** The antioxidant activity of hydrogels.

Method		HEa-EtOH	HEa-iPrOH	HEa-WA	H-CON
DPPH	mg Trolox/g hydrogel	3.43 ± 0.01^a^	3.52 ± 0.02^a^	3.15 ± 0.01^a^	na
ABTS		10.05 ± 0.19^b^	12.35 ± 0.28^c^	7.58 ± 0.30^a^	na
TC	mg GA/g hydrogel	9.02 ± 0.14^b^	11.43 ± 0.14^c^	4.89 ± 0.29^a^	na

DPPH, antioxidant activity measured using 2,2-diphenyl-1-picrylhydrazyl; ABTS, antioxidant activity measured using 2,2′-azinobis (3-ethylbenzothiazoline-6-sulfonic acid); TC, total polyphenol content; GA, gallic acid; HEa-EtOH, hydrogel containing dry ethanolic extract of *E. angustifolium*; HEa-iPrOH, hydrogel containing dry isopropanol extract of *E. angustifolium*; HEa-WA, hydrogel containing dry water extract of *E. angustifolium;* H–CON, hydrogel without *E. angustifolium* extract. na—no activity. Different letters indicate significant differences between the HEas, α = 0.05. Values are the mean of three replicate determinations (*n* = 3).

#### 3.3.2 Determination of Anti-inflammatory Properties

**FIGURE 3:**
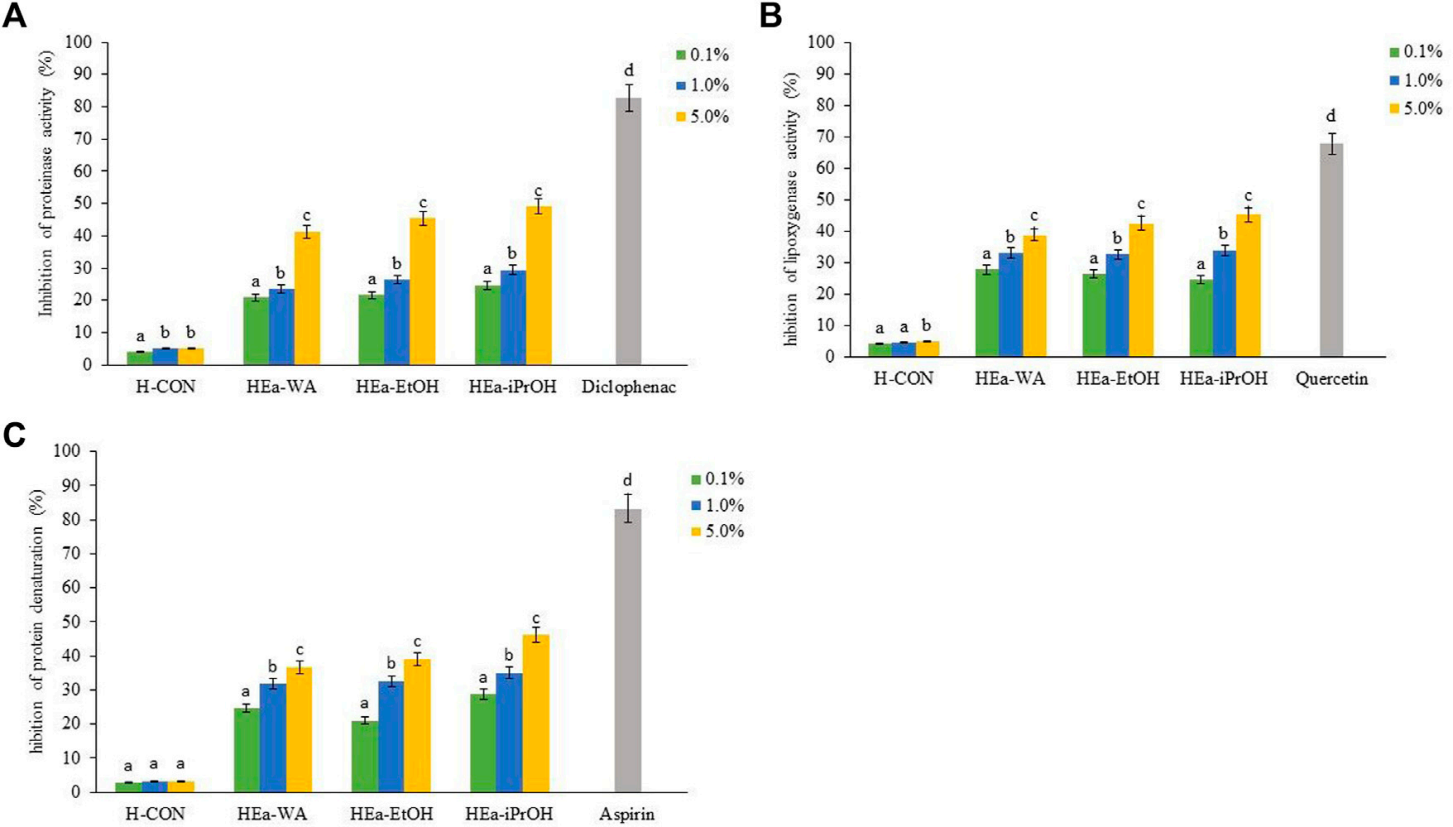
Anti-Inflammatory properties of HEas. The proteinase inhibition **(A)**, the lipoxygenase inhibition **(B)**, the inhibition of protein denaturation **(C)**. HEa-EtOH—hydrogel containing dry ethanolic extract of *E. angustifolium*; HEa-iPrOH—hydrogel containing dry isopropanol extract of *E. angustifolium*; HEa-WA—hydrogel containing dry water extract of *E. angustifolium*; (H)CON—hydrogel without *E. angustifolium* extract. All hydrogels were used in 0.1, 1.0 and 5.0%. Diclofenac **(A)**, quercetin **(B)** and aspirin **(C)** was used as a control inhibitor. Different letters indicate significant differences between the HEas, α = 0.05. Values are the mean of three replicate determinations (*n* = 3).

The results regarding the anti-inflammatory properties of HEas are shown in Figure 3. The obtained results indicate the dose-dependent anti-inflammatory activity of the hydrogels tested. The most potent anti-inflammatory properties were obtained for HEa-iPrOH > HEa-EtOH > HEa-WA. The most favourable of the tested concentrations was 5.0%, for which a reduction in the activity of enzymes lipoxygenase and proteinase as well as inhibition of protein denaturation, reaching almost 50% for the HEa-iPrOH was achieved (Figure 3).

#### 3.3.3 Biocompatibility Study

**FIGURE 4:**
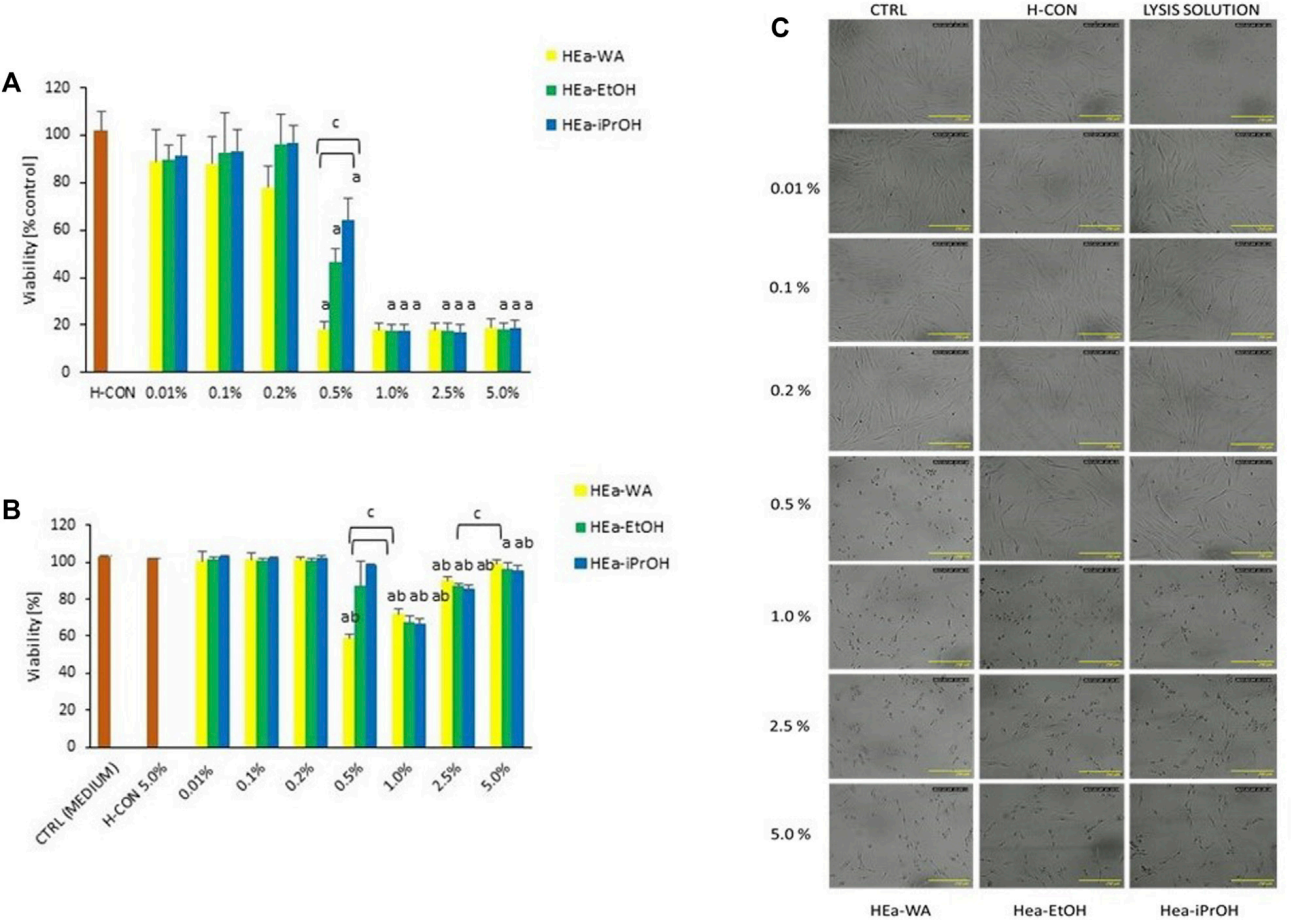
The primary human fibroblasts viability determined using PrestoBlue assay **(A)** and LDH assay **(B)** after 24 h of treatment with: 0.01, 0.1, 0.2, 0.5, 1.0, 2.5, and 5.0% of the tested hydrogels corresponding to 5, 50, 100, 250, 500, 1250, and 2,500 μg/ml of *E. angustifolium* extracts. The results are expressed as the mean and SD from three independent experiments; a = *p* < 0.05 vs. H-CON; b = *p* < 0.05 vs. ctrl; c = *p* < 0.05 vs. HEa-WA. Optical microscopy images of primary human fibroblasts after 24 h incubation with the standard medium (negative control), 5.0% of hydrogel without HEas (as vehicle control, H-CON), Lysis Solution (positive control in LDH assay) and 0.01, 0.1, 0.2, 0.5, 1.0, 2.5, and 5.0% of the tested hydrogels (HEa-WA, HEa-EtOH and HEa-iPrOH) corresponding to 5, 50, 100, 250, 500, 1250, and 2,500 μg/ml of *E. angustifolium* extracts. Cells with the medium were used as a negative control, and cells treated with the hydrogel (5%) without HEas were used as a vehicle control (H-CON) **(C)**.

The biocompatibility of tested hydrogels was evaluated in primary human fibroblasts in relation to the metabolic activity (PrestoBlue assay) and cell membrane permeability (LDH assay) (Figure 4). After 24 h of incubation, both assays confirmed the biocompatibility of pure vehicle—hydrogel without HEas, even at the highest concentration tested (5.0%). However, both methods revealed that all formulations containing 500–2,500 μg/ml of *E. angustifolium* extracts significantly decreased cell viability below 20% and 66%, according to the PrestoBlue and LDH assays, respectively. Hence, the obtained findings suggest that the differences in cell metabolic activity were more distinct than membrane damage related to cell death after 24 h of incubation.

The most striking differences in HEas cytotoxicity were observed at 0.5% (250 μg/ml of *E. angustifolium* extracts) where fibroblasts viability decreased to 18.27%/58.64%,46.26%/87.10%, and 64.12%/98.04% in response to HEa-WA, HEa-EtOH, and HEa-iPrOH, respectively, according to PrestoBlue/LDH assay. At the hydrogels concentrations between 0.01 and 0.2% only PrestoBlue showed further but less significant differences in cells viability. The obtained results were consistent with the microscopy imaging (Figure 4C) where starting from the hydrogels of 0.2% no differences in the cell morphology between HEa-WA/HEa-EtOH/HEa-iPrOH-treated and control cells were detected.

#### 3.3.4 Wound Assay

**FIGURE 5:**
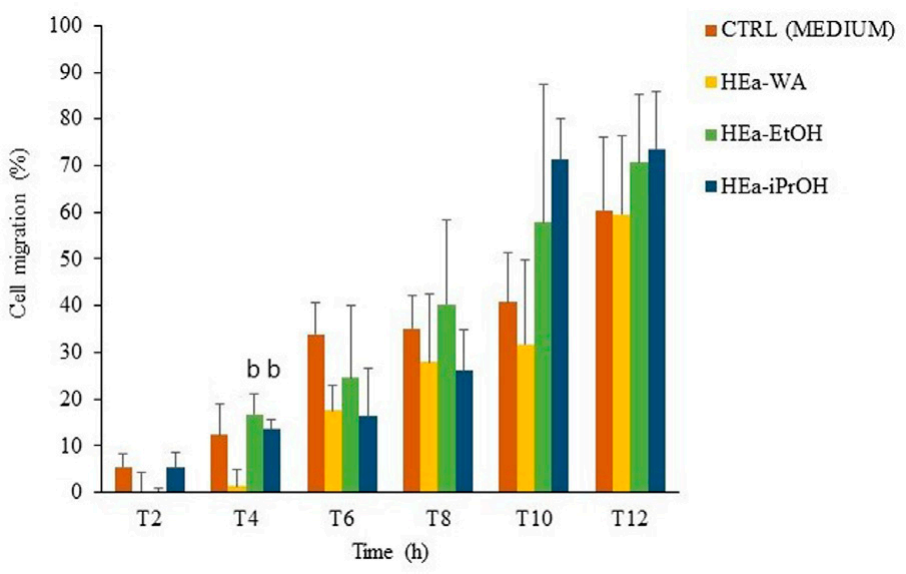
Wound healing assay activity of the hydrogels at 0.2% (100 μg/ml of *E. angustifolium* extracts) and medium as a negative control. The mean and SD of the cell migration from three independent experiments is shown: b = *p* < 0.05 vs. HEa-WA.

The highest biocompatible concentration for all formulations, i.e. 0.2% 100 μg/ml of EA, was chosen to measure wound healing activities as important for repairing new tissues. As shown in Figure 5, the most significant differences in hydrogels activity were observed at 4 h, where surprisingly HEa-WA inhibited cell mobility. Moreover, in general, the presence of alcohol extract seemed to accelerate fibroblasts migration (Supplementary Figure S2; Supplementary Movies S3–S6). Namely, HEa-EtOH and HEa-iPrOH enhanced wound healing activity of HDF cells to 57.78% and 71.33% in contrary to HEa-WA (31.71%) and control (40.72%) at 10 h. Still, the observed differences were not statistically significant After 12 h of treatment, the differences in wound closure became less distinct. Approximately 60%–70% of the gap was closed for CTRL/HEa-Wa and HEa-EtOH/HEa-iPrOH, respectively.

#### 3.3.5 Microbiological Activity

The response of microorganisms to the individual HEas was similar, and the differences between the unique hydrogels were not statistically significant (Table 4). However, different reactions between particular strains of bacteria were observed. The most considerable inhibition zone was observed for *S. pneumoniae,* followed by *E. coli, E. faecalis* *, E. faecium, S. lutea* and *B. pseudomycoides,* while the strains of *S. epidermidis*, *B. subtilis* and *S. aureus* were the least sensitive (Table 4).

**TABLE 4:** Effect of all hydrogels on inhibiting the growth of selected bacteria.

Strain of bacteria	HEa-WA	HEa-EtOH	HEa-iPrOH	H-CON
	The diameter of the growth inhibition zone (mm)			
*Escherichia coli*	13.0 ± 0.5^a^	15.0 ± 0.5^a^	14.0 ± 1.0^a^	nr
*Enterococcus faecalis*	16.0 ± 0.5^a^	17.0 ± 0.5^a^	17.0 ± 0.5^a^	nr
*Enterococcus faecium*	14.5 ± 1.5^a^	16.0 ± 0.5^a^	16.0 ± 1.5^a^	nr
*Sarcina lutea*	15.0 ± 1.5^a^	16.5 ± 0.5^a^	16.5 ± 0.5^a^	nr
*Serratia marcescens*	9.0 ± 0.5^a^	10.5 ± 0.5^a^	10.0 ± 0.5^a^	nr
*Pseudomnas fluorescens*	8.0 ± 1.0^a^	8.5 ± 1.0^a^	7.0 ± 0.5^a^	nr
*Streptococcus pnemoniae*	16.5 ± 1.0^a^	18.0 ± 0.5^a^	17.0 ± 0.5^a^	nr
*Bacillus subtilis*	6.5 ± 0.5^a^	8.0 ± 0.5^a^	8.5 ± 1.0^a^	nr
*Bacillus pseudomycoides*	10.0 ± 0.5^a^	13.0 ± 0.5^a^	13.0 ± 1.5^a^	nr
*Staphylococcus aureus*	6.0 ± 0.5^a^	7.0 ± 0.5^a^	7.0 ± 0.5^a^	nr
*Streptococcus epidermidis*	6.0 ± 1.0^a^	8.5 ± 0.5^a^	7.0 ± 0.5^a^	nr

nr, no reaction.

### 3.4 Permeation Through the Skin

**FIGURE 6:**
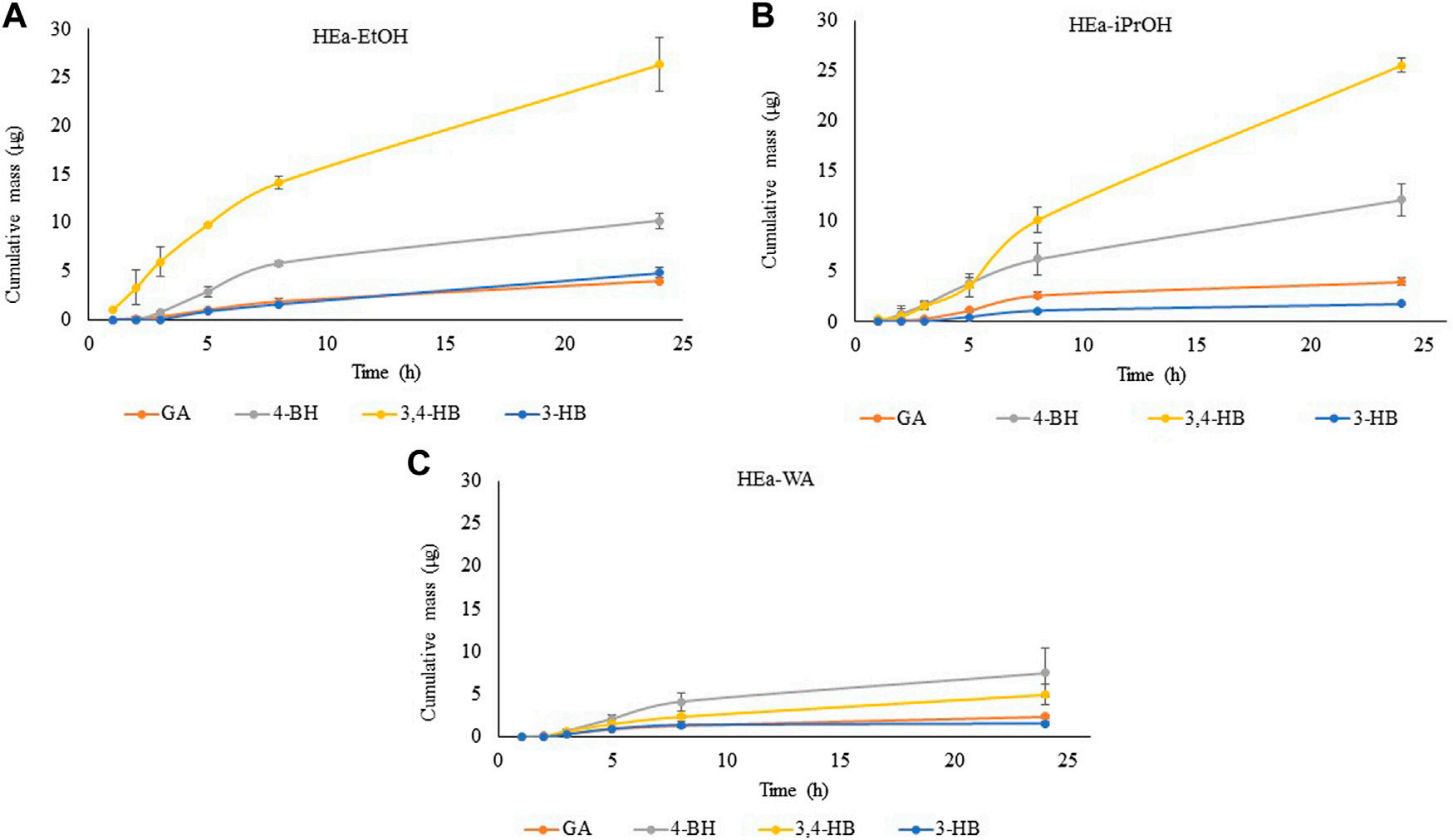
Time course of the cumulative mass of phenolic acids during the 24 h permeation. In the donor chamber placed 1 cm^3^ of HEas; HEa-EtOH—hydrogel containing dry ethanolic extract of *E. angustifolium* **(A)**; HEa-iPrOH—hydrogel containing dry isopropanol extract of *E. angustifolium* **(B)**; HEa-WA—hydrogel containing dry water extract of *E. angustifolium* **(C)**; Values are expressed as mean ± SD (*n* = 3).

The parameters of permeation of phenolic acids during the 24-h experiment are shown in Table 5. The cumulative mass in acceptor fluid, considering all time points, is presented in Figure 6. All EA extracts incorporated in hydrogels permeated the pigskin into the acceptor phase solutions. Generally, the highest permeation of phenolic acids were demonstrated for HEa-EtOH and HEa-iPrOH, while lower from HEa-WA. The phenolic acids permeated through pigskin at different rates depending on the hydrogel used. The highest permeation parameters were observed for HEa-iPrOH. The highest permeation rate of 1.33, 0.90, 0.54, 0.39, and 0.22 μg cm^−2^ h^−1^, were observed for 3,4-HB, 4-HB, GA, and 3-HB from HEa-iPrOH, respectively (Figure 6; Table 5).

**TABLE 5:** The parameters characterize phenolic acids transport through the skin after applying HEas in the penetration study.

Phenolic acid	C_m_	J_ss_, μg/cm^2^ h^1^	K_P_·10^3^, cm/h	L_T,_ h	D∙10^4^, cm^2^/h	K_m_	Q%_24 h_
	HEa-EtOH						
GA	3.97 ± 0.12	0.27 ± 0.05	8.17 ± 0.17	1.45 ± 0.06	2.86 ± 0.12	1.42 ± 0.36	11.76 ± 0.36
4-HB	10.17 ± 0.80	0.88 ± 0.03	7.93 ± 0.30	1.67 ± 0.10	2.49 ± 0.01	1.59 ± 0.13	9.16 ± 0.72
3,4-DHB	26.27 ± 2.77	1.81 ± 0.11	8.63 ± 0.53	0.09 ± 0.00	45.18 ± 1.65	0.09 ± 0.00	12.52 ± 1.32
3-HB	4.83 ± 0.51	0.31 ± 0.04	1.93 ± 0.02	2.74 ± 0.49	1.51 ± 0.40	0.63 ± 0.17	2.99 ± 0.31
	HEa-iPrOH						
GA	3.98 ± 0.38	0.38 ± 0.03	15.75 ± 1.60	1.60 ± 0.18	2.58 ± 0.33	3.04 ± 0.64	16.28 ± 1.58
4-HB	12.15 ± 1.57	0.90 ± 0.02	7.62 ± 0.19	0.92 ± 0.03	4.23 ± 0.15	0.89 ± 0.05	10.25 ± 1.33
3,4-DHB	25.50 ± 0.68	1.32 ± 0.17	6.18 ± 0.80	0.00 ± 0.00	16.08 ± 2.59	0.01 ± 0.00	11.88 ± 0.31
3-HB	1.78 ± 0.06	0.22 ± 0.00	2.53 ± 0.28	2.95 ± 0.19	1.40 ± 0.08	0.90 ± 0.01	2.05 ± 0.07
	HEa-WA						
GA	2.34 ± 0.22^a^	0.20 ± 0.02^a^	8.20 ± 0.11^a^	1.03 ± 0.14^a^	4.01 ± 0.53^a^	1.01 ± 0.53^a^	9.56 ± 0.93^a^
4-HB	7.49 ± 2.89	0.69 ± 0.18	7.16 ± 0.18	1.99 ± 0.40	2.08 ± 0.51	1.71 ± 0.69	8.22 ± 0.72
3,4-DHB	4.96 ± 1.24	0.36 ± 0.02	6.36 ± 0.49	1.34 ± 0.18	3.10 ± 0.42	1.02 ± 0.20	8.70 ± 0.21
3-HB	1.56 ± 0.18	0.21 ± 0.06	4.96 ± 0.14	1.78 ± 0.40	3.88 ± 0.62	1.51 ± 0.57	3.75 ± 0.44

GA, gallic acid; 3,4-DHB, 3,4-dihydroxybenzoic acid; 4-HB, 4-hydroxybenzoic acid; 3-HB, 3-hydroxybenzoic acid; HEa-EtOH, hydrogel containing dry ethanolic extract of *E. angustifolium*; HEa-iPrOH, hydrogel containing dry isopropanol extract of *E. angustifolium*; HEa-WA, hydrogel containing dry water extract of *E. angustifolium*; C_m_, cumulated mass after 24-h penetration, J_SS_, steady-state flux; K_P_, permeability coefficient; L_T_, lag time; D, diffusion coefficient; K_m_, skin partition coefficient; Q%_24 h_—the percentage of the applied dose after 24 h; Values are expressed as mean ± SD (*n* = 3).

### 3.5 Accumulation in the Skin

**FIGURE 7:**
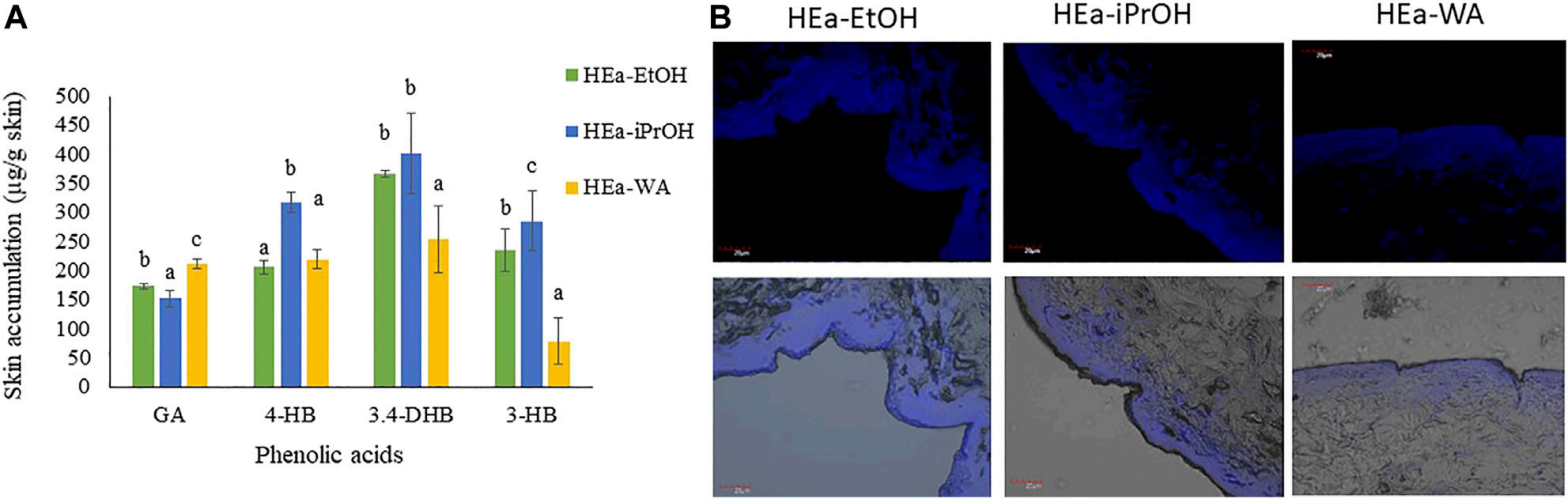
Accumulation in the skin of phenolic acids after 24-penetration; different letters indicate significant differences between the HEas, α = 0.05 **(A)**. The microscopic photos of vertical slicing of porcine pig skin sections 24 h after applying the HEas. The polyphenols are visible in the upper layer of the skin, along the SC. Visible are the polyphenols blue under a fluorescence effect (blue color) **(B)**. HEa-EtOH—hydrogel containing dry ethanolic extract of *E.* angustifolium; HEa-iPrOH—hydrogel containing dry isopropanol extract of *E.* angustifolium, HEa-WA-hydrogel containing dry water extract of *E. angustifolium*.

The accumulation of individual phenolic acids in the skin after 24-h penetration is shown in Figure 7. After application, the all of the hydrogels the accumulation of phenolic acids in the skin was observed. However, accumulation was varied depending on the hydrogel used. The highest accumulation of 3,4-DHB, 4-HB, and 3-HB were found by the skin after HEa-iPrOH application, where the content of these acids was 401.42 ± 51.67; 316.79 ± 17.36, and 285.40 ± 2.92 μg/g skin, respectively. On the other hand, GA accumulated in the most significant amount after the application on the skin of HEa-EtOH, reaching the value 173.64 ± 11.81 μg/g skin—Figure 7A. Images from transverse sections of pig skin after application of all hydrogels are shown in Figure 7B. It has been observed that polyphenols strongly accumulate mainly along with the SC of the skin. In images, marked with an intense fluorescence along with *stratum corneum* (SC). Pictures obtained with control pigskin, where the H-CON solution was applied, did not show any fluorescence—Figure 7B.

**FIGURE 8:**
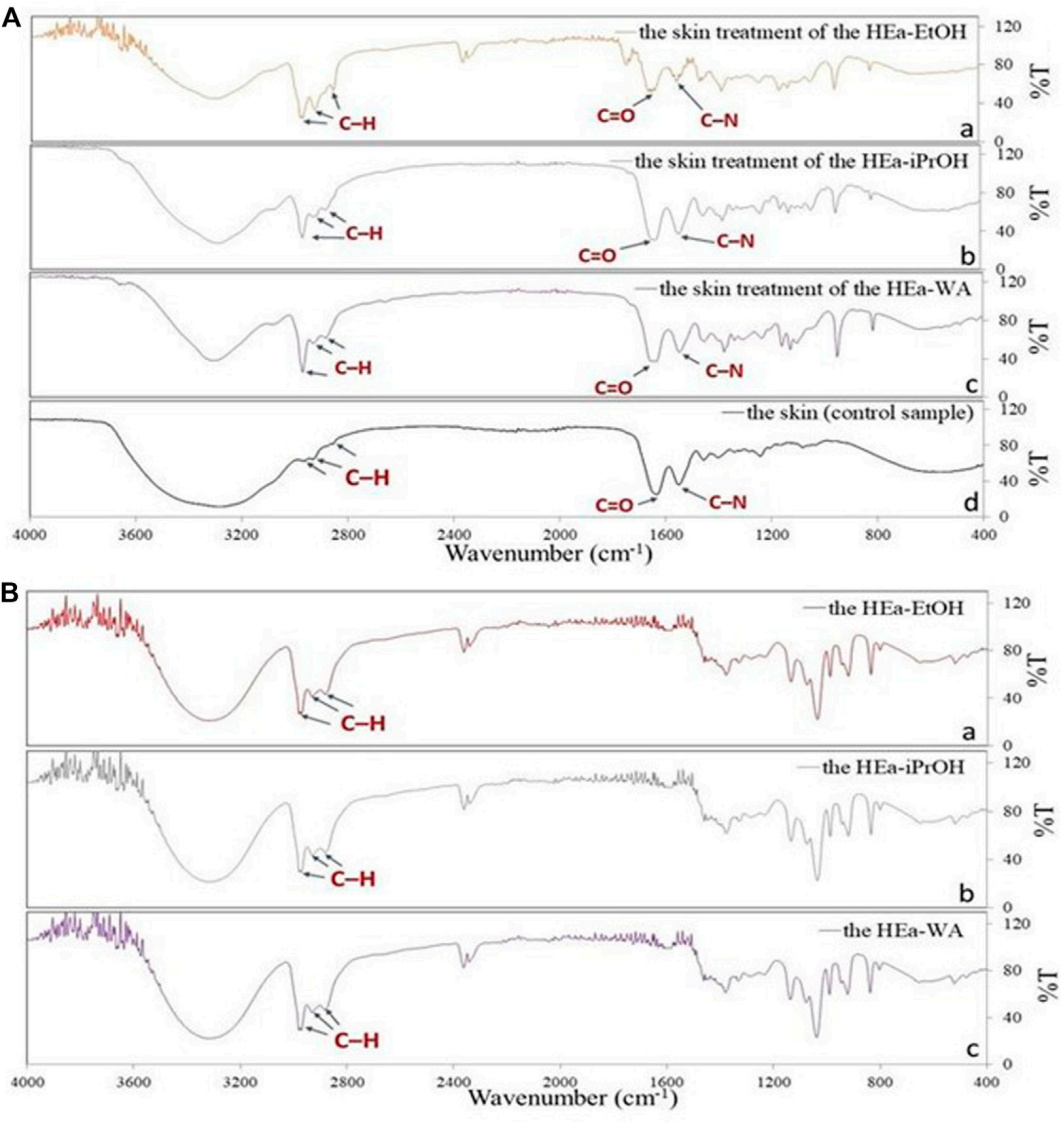
ATR-FTIR absorption spectra of **(a)** pigskin treated with the hydrogel containing extract of Ea-EtOH; **(b)** pigskin treated with the hydrogel containing extract of Ea-iPrOH; **(c)** pigskin treated with the hydrogel containing extract of Ea-WA; **(d)** pure pigskin (control sample) **(A)**; ATR-FTIR absorption spectra of **(a)** the hydrogel containing extract of Ea-EtOH; **(b)** the hydrogel containing extract of Ea-iPrOH; **(c)** the hydrogel containing extract of Ea-WA **(B)**. HEa-EtOH—hydrogel containing dry ethanolic extract of *E. angustifolium*; HEa-iPrOH—hydrogel containing dry isopropanol extract of *E. angustifolium*; HEa-WA—hydrogel containing dry water extract of *E. angustifolium*.

The FTIR spectra of the skin treated with the hydrogel containing HEas were presented in Figure 8A. The spectrum shows intensive peak (2,970 cm^−1^) and small peaks (2,930 and 2,880 cm^−1^) of the C–H of the alkyl groups. No intense peak was observed in the control sample. i.e. skin without application of HEas (Figure 8B).

## 4 Discussion

Recently, more and more attention has been paid to use plant extracts applied to the skin in various types of vehicles, including hydrogels. The hydrogels play an increasingly important role in medicine, pharmacy and cosmetology. In recent years, hydrogel formulations with natural ingredients have gained significant attention for treating various dermatological disease (Fibrich et al., 2020; Zagorska-Dziok and Sobczak, 2020). They are characterized by many positive features: biocompatibility, non-toxicity, bifunctionality, and biodegradability (Zagorska-Dziok and Sobczak, 2020). In addition, the hydrogels containing plant extracts can have a wide range of effects, including antioxidant, antibacterial and anti-inflammatory and can be an excellent vehicle for active substances found in plants (Zagorska-Dziok and Sobczak, 2020; Michalak et al., 2021). In our study, we assessed the biological activity of the hydrogels containing extracts of *E. angustifolium*. In addition, we also evaluated the permeation through the pigskin and accumulation in the skin of the phenolic acids contained in this plant.

The estimated content of secondary metabolites in natural preparations can be helpful to assess their potential pharmacological activity. One of the group of compounds that play a crucial role in the effects of preparations applied to the skin are phenolic acids belonging to a large group of polyphenols. The phenolic acids are valuable compounds due to their, among others, antioxidant, antibacterial, anticarcinogenic, and anti-inflammatory properties. In our study, phenolic acids, such as GA, 3,4-DHB, 3-HB, and 4-HB were identified in all hydrogels containing *E. angustifolium* extracts*.* Our previous research identified such compounds in the 70% (v/v) ethanol extract of *E. angustifolium* (Nowak et al., 2021a; 2021d). Similarly, some phenolic acids, like GA, 3,4-DHB, 4-HB, and 3-HB, were also found in the extracts of *E. angustifolium* by Ruszova et al., Zagorska-Dziok et al. and Lasinskas et al. (Ruszova et al., 2014; Lasinskas et al., 2020; Zagorska-Dziok et al., 2021b). Due to their high antioxidant activity, polyphenols are considered to be crucial in skin protection against oxidative stress. The skin is constantly exposed to oxidative stress induced by reactive oxygen species (ROS), damaging cellular constituents, such as DNA, cell membrane lipids or proteins. To protect skin against the harmful effect of free radicals, the skin is equipped with antioxidant defense mechanisms (Briganti and Picardo, 2003). Therefore, it is very important to support the body’s endogenous protective system by exogenous antioxidants included in plants. Many studies report high antioxidant activity of *E. angustifolium* extracts (Shikov et al., 2006; Onar et al., 2012; Ruszová et al., 2014; Nowak et al., 2019; Jariene et al., 2020; Lasinskas et al., 2020; Nowak et al., 2021d, 2021a). In our study, all the hydrogels containing *E. angustifolium* extracts used were characterized by antioxidant activity. However, it turns out that the type of extract added to hydrogels was also of importance. The highest scavenging free radicals were the HEa-iPrOH, the next HEa-EtOH showed, and the lowest HEa-WA. The difference in antioxidant activity between various extracts from *E. angustifolium* was reported in earlier studies. The highest antioxidant activity for *E. angustifolium* in the flowering phase was observed for extracts in 70% ethanol as well as for extracts in 70%, isopropanol, and the lowest for water extracts (Nowak et al., 2019). The low levels of reactive oxygen species (ROS) are conducive to normal wound healing by stimulating cell migration and angiogenesis. However, excessive ROS can hinder wound healing, especially in chronic wounds (Xu et al., 2020). Therefore, the introduction of natural antioxidants may be very effective in this case. Many studies report faster healing of wounds due to the high antioxidant activity of plants (Singh et al., 2006; Karakaya et al., 2020). For example, in rats by *Aristolochia bracteolate* ethanolic dried leaf extract was correlated with the increase of two potent antioxidant enzymes such as superoxide dismutase and catalase in granuloma tissue (Shirwaikar et al., 2003). The hydrogel with antioxidant activity, applied to the skin can reduce oxidative stress, improve the wound microenvironment, and ultimately achieve rapid skin repair (Xu et al., 2020).

Bacterial infection is one of the key obstacles to the wound healing process. Many pathogens are associated with skin infections. For example, Gram-positive *staphylococci* and *streptococci* cause wound infections, boils, carbuncles, abscesses, impetigo and erysipelas. The Gram-negative *Enterobacteria* may cause wound infections and sepsis. A frequent pathogen of wound infections is the *Pseudomonas* (Weckesser et al., 2007). In these cases, more and more popular the hydrogels show, among others, antibacterial activity (Zhang et al., 2021). The secondary metabolites of plants, mainly polyphenols, characterized by a strong antibacterial effect, play an important role here (Slobodníková et al., 2016). Therefore, the next step in our study was to evaluate the antibacterial activity of the HEas. All the hydrogels used showed a similar antibacterial effect but it depended on the bacterial strain. The bacteria *S. pneumoniae* turned out to be the most sensitive, either a skin colonizer or soft tissues. As a pathogen, it causes clinical diseases that vary widely in prognosis and severity (Newman et al., 2005; Garcia-Lechuz et al., 2007). Generally, the herb *E. angustifolium* showed solid antibacterial properties (Battinelli et al., 2001; Kosalec et al., 2008; Ferrante et al., 2020; Nowak et al., 2021a). Therefore, in the face of increasing antibiotic resistance, the inclusion of this plant could be an exciting alternative to “synthetic” preparations used topically in the treatment of infected wounds.

Inflammation of the skin is a complex biological response to external factors such as, among others, pathogens and irritants (Fibrich et al., 2020). Natural plant compounds can replace synthetic anti-inflammatory drugs in combating inflammatory conditions’ causes and effects (Oguntibeju, 2018). Generally, it is believed that natural preparations are characterized by much greater safety and fewer side effects, while their use may become an effective treatment strategy for chronic inflammation (Azab et al., 2016). Therefore, in our study, the anti-inflammatory properties of the HEas were investigated by assessing the possibility of inhibiting lipoxygenase, proteinase activity and the effect on protein denaturation. Commonly known compounds with anti-inflammatory properties such as diclofenac, quercetin and aspirin were used as controls. Lipoxygenase is an oxidative enzyme with an active non-heme iron atom and it regulates the inflammatory response (Wisastra and Dekker, 2014). These enzymes catalyze the oxidation of polyunsaturated fatty acids such as linoleic and arachidonic acids. The effect of lipid oxidation is the commencement of the biological reactions and the activation of various cell signaling mechanisms (Mashima and Okuyama, 2015).

In contrast, the proteinases affect the degradation of skin proteins, such as collagen and elastin, which, among others, determine its flexibility (Ziemlewska et al., 2021). An important parameter assessing the anti-inflammatory properties of plant material is protein denaturation. Protein denaturation significantly affects its spatial structure and the loss of its biological properties, contributing to various inflammatory diseases. Therefore, the ability of plant extracts to prevent protein denaturation may also help to prevent inflammation (Nowak et al., 2021d). All analyzed in our study hydrogels exhibited anti-inflammatory effects in our study, with the best impact observed for HEa-iPrOH. The most favourable of the tested concentrations was 5.0%, for which a reduction in the activity of enzymes and inhibition of protein denaturation reached almost 50%. The ability to inhibit lipoxygenase and denature proteins depending on the concentration used confirmed the results at previous research assessing the ethanol extracts (Nowak et al., 2021d) and water extract (Onar et al., 2012) with *E. angustifolium*. The analyzed in our study hydrogels have a lower anti-inflammatory activity than commonly known anti-inflammatory compounds. However, the obtained values of inhibition of BSA denaturation and reduction of the activity of tested enzymes indicate the potential of their use in the treatment of inflammatory skin diseases.

According to ISO 10993-5:2009 “Biological evaluation of medical devices” substances are biocompatible if they do not diminish cell viability below 75% (Chitra et al., 2019). Therefore in the current study, regardless of the extraction method (and viability assay), hydrogels containing 5–100 μg/ml of the EA extracts may be considered non-toxic and appropriate for biomedical applications. Our data are in line with Kiss et al. study, where extracts of the three most popular *Epilobium* species (*E. angustifolium*, *E. hirsutum* and *E. parviflorum*) at concentrations 3.125–50 μg/ml demonstrated no significant cytotoxic activity on human skin fibroblast cells (Kiss et al., 2011). Cell proliferation and cell migration to the site of tissue damage are necessary for effective wound healing (Srirod and Tewtrakul, 2019). The current study revealed that tested hydrogels, HEa-EtOH and HEa-iPrOH but not HEa-WA, slightly improve fibroblasts migration after scratch within the first 10 h. However, the obtained data are not in line with Sudsai et al. study where the EtOH extract and CHCl_3_ fraction of *B. longiflora* significantly enhanced L929 fibroblast migration (Sudsai et al., 2013). Moreover, according to Ruszova et al., isopropanol extract of *E. angustifolium* revealed a protective effect on the viability of senescent NHDF induced by serum deprivation (Ruszová et al., 2014). Our wound healing assay findings are in keeping with phenolic content and antioxidant activity study, where the most favourable effect was observed as follows: HEa-iPrOH > HEa-EtOH > HEa-WA. The high antioxidant activity of hydrogels applied to the skin may play an essential role in wound healing (Xu et al., 2020) because inhibiting excessive production of free radicals contribute to a faster therapeutic effect (Ali et al., 2020). According to Karakaya et al., the EtOAc sub-extract of the aerial part of *E. angustifolium* displayed remarkable wound healing activity related to high antioxidant activities and hyperoside content (Karakaya et al., 2020).

The biological activity of preparations applied to the skin depends primarily on the permeation of the most important active substances. In the case of topical penetration, it is very important to release the given substances from the preparation form and to reach all layers of the skin as well as the underlying layers (Bertges et al., 2020). The hydrogels can be an excellent vehicle for drugs for topical application (Ali et al., 2020; Zagórska-Dziok and Sobczak, 2020), and the additional inclusion of the plant in the hydrogel can enrich the skin with valuable active substances. The active ingredients derived from plants included in the hydrogel can permeate through the skin or accumulate in it. In our previous studies, we have shown the permeation of phenolic acids from extract in 70% (v/v) ethanol (Nowak et al., 2021a) and from dry ethanol extracts incorporated into hydrogel and emulsion (2,5% and 5% (w/w)) (Nowak et al., 2021d) as well as extracts in 70% (v/v) ethanol extracts incorporated in bacterial cellulose membranes (Nowak et al., 2021c). However, it is known that the method of preparing the extract itself, and the choice of the solvent, for extraction plays a significant role in isolating active substances from plants. Therefore, the next step of our research was to estimate the permeation through the pigskin of selected phenolic acids from three hydrogels, differing in the solvent used to prepare plant extract. Then we prepared three hydrogels containing dry plant extracts at a concentration of 5%. Incorporating plant extracts at concentrations above 5% into the vehicle could lead to physical instability of the formulation (Bertges et al., 2020). Our research showed that all plant extracts in hydrogels permeated the skin into the acceptor phase solution. The highest permeation of the phenolic acids was generally demonstrated after HEa-EtOH and HEa-iPrOH, while the much lower for HEa-WA. Chromatographic analysis showed that the extracts obtained with alcohols as solvents are characterized by the highest content of phenolic acids, which was reflected in greater permeation. The permeation of phenolic acids from the various vehicles through the skin was confirmed in other studies. For example, Žilius et al. showed the high permeation of phenolic acids, such as coumaric, caffeic, and ferulic, from a hydrogel containing a propolis extract (Žilius et al., 2013). On the other hand, in other studies the penetration of chlorogenic acid from the hydrogel containing 5% (w/w) coffee extract (Bertges et al., 2020) and from the hydrogel containing 5% (w/w) *Viscum album* extract was observed (Batista et al., 2021). The other penetration of polyphenols and other plant compounds through the skin can be affect by many factors. Numerous studies have shown that polyphenolic compounds exhibit differences in permeation capacity. The composition of the formulation is a significant factor. The results of our research on the penetration of phenolic acids was likely to be exerted by the moisturizing ingredient of the preparation, such as propylene glycol, which enhance membrane permeability by acting as an absorption promoter (Ratz-Łyko et al., 2015; Batista et al., 2021). Greater delivery of the active substance to the skin increases the effect of cosmetic and dermatological preparations. On the other hand, the accumulation of active substances in the skin is also very desirable, especially in the case of preparations having a local effect (Nicolai et al., 2020). In these cases, the ingredients penetrating deep into the skin act impact as long as possible. In our study, the accumulation of polyphenols in the skin was also observed. The fluorescence method shows that these compounds were primarily accumulated in the skin’s upper layers across the SC. The fluorescence method is a good alternative for identifying active substances, and evaluating the depth of penetration (Martimestres et al., 2007). However, since all polyphenols from fluorescent derivatives with the 2-aminoethyl diphenyl borinate reagent, it would be difficult to identify specific phenolic acids. Therefore, our study identified the total pool of polyphenols accumulated in the skin. Many authors confirm the accumulation of polyphenols in of the skin (Martimestres et al., 2007; Alonso et al., 2014, 2015; Batista et al., 2021). The difference between the intensity of C–H bonds of the skin treated of the HEas and control sample was observed. In our study, in the case of the skin treated with the HEas, the spectrum shows intensive peak (2,970 cm^−1^) and small peaks (2,930 and 2,880 cm^−1^) coming from the C–H of the alkyl groups. No intense peak of the C–H of the alkyl groups was observed in the control sample—skin without application of HEas. FTIR of the control sample (skin without application of HEas) showed weak peaks at 2,960, 2,930, and 2,880 cm^−1^, which are attributed to the C-H groups derived from cholesterol, long alkyl chains of ceramides or fatty acids, and which are the main components of SC lipids (Ahad et al., 2016). In FTIR analysis we also observed the shift peaks C-N. A shift of the peaks C-N stretching vibration may be due to the fact that the SC is composed of lipids (ceramides) that are tightly packed, so the penetration of some ingredients contained in the hydrogel into the SC lipid bilayers leads to the disruption of the hydrogen bond network at the head of ceramides (Jain, 2002; Amnuaikit et al., 2005). Our studies confirmed that the active substances derived from *E. anustifolium* are absorbed into the skin. FTIR of the skin treated with the HEas showed that the characteristic peaks of derived from hydrogel components appear without any change in the spectrum of the skin. These results indicated that there was no interaction to be detected between the skin and the components of the hydrogel (Hussein et al., 2016). Performing studies to confirm that active substances accumulate in the skin may be helpful in the design of dermatological or cosmetic preparations, the main task of which is multiple local effects (Hussein et al., 2016; Ahad et al., 2016; Jiang et al., 2017; Nowak et al., 2021b).

## 5 Conclusion

Nowadays, more and more attention is drawn to natural preparations that are easy to apply, safe and show a wide therapeutic action (Žilius et al., 2013). However, despite the extensive knowledge passed down “for generations” on the use of *E. angustifolium* and its current use in many world regions, there are not many standardized preparations containing this plant. The results of our study confirm the possibility of using *E. angustifolium* as a component of dermatological and cosmetic preparations by influencing some processes causing skin changes, namely: oxidative stress, bacterial infections or inflammations. The secondary metabolites that occur in this plant in large amounts are primarily responsible for this action, such as polyphenols, which primarily responsible for reducing oxidative stress as well as that may have an impact on faster wound healing and anti-aging effect. The terpenes in HEAs, on the other hand, can play an important role in fighting bacterial skin infections. Additionaly, the hydrogels containing alcoholic extracts, i.e., HEa-EtOH and HEa-iPrOH, were the most beneficial compared to HE-WA, which can be valuable information regarding the choice of solvent for the preparation of the plant material. It should be pointed that the biocompatibility studies have shown the necessity to limit the concentration of EA to 100 μg/ml, which is probably due to the complex chemical composition of plant extracts. In the future, it is planned to determine the compounds responsible for the desired biological properties and to contain these selected active substances in hydrogels. Summarize, the hydrogels containing alcoholic extracts of EA may be an interesting for topical products for skin care and treatment.

## Data Availability

The original contributions presented in the study are included in the article/Supplementary Material, further inquiries can be directed to the corresponding author.

## Supplementary Material

Supplementary Video S1 CTRL.

Supplementary Video S2 HEa-iPrOH.

Supplementary Video S3 HEa-EtOH.

Supplementary Video S4 HEa-WA.
