# Corrigendum: Assessment of the anti-inflammatory, antibacterial and anti-aging properties and possible use on the skin of hydrogels containing *Epilobium angustifolium* L. extracts

**DOI:** 10.3389/fphar.2022.991766

**Published:** 2022-08-12

**Authors:** Anna Nowak, Martyna Zagórska-Dziok, Magdalena Perużyńska, Krystyna Cybulska, Edyta Kucharska, Paula Ossowicz-Rupniewska, Katarzyna Piotrowska, Wiktoria Duchnik, Łukasz Kucharski, Tadeusz Sulikowski, Marek Droździk, Adam Klimowicz

**Affiliations:** ^1^ Department of Cosmetic and Pharmaceutical Chemistry, Pomeranian Medical University in Szczecin, Szczecin, Poland; ^2^ Department of Technology of Cosmetic and Pharmaceutical Products, Medical College, University of Information Technology and Management in Rzeszow, Rzeszów, Poland; ^3^ Department of Experimental and Clinical Pharmacology, Pomeranian Medical University in Szczecin, Szczecin, Poland; ^4^ Department of Microbiology and Environmental Chemistry, Faculty of Environmental Management and Agriculture, West Pomeranian University of Technology, Szczecin, Poland; ^5^ Department of Chemical Organic Technology and Polymeric Materials, Faculty of Chemical Technology and Engineering, West Pomeranian University of Technology, Szczecin, Poland; ^6^ Department of Physiology, Pomeranian Medical University in Szczecin, Szczecin, Poland; ^7^ Department of Pharmaceutical Chemistry, Pomeranian Medical University, Szczecin, Poland; ^8^ Clinic of General Surgery, Minimally Invasive and Gastrointestinal, Pomeranian Medical University in Szczecin, Szczecin, Poland

**Keywords:** *E. angustifolium*, hydrogels, antioxidant, skin penetration, anti-aging, wound healing, anti-infammatory

In the published article, there was an error in Figure 7B as published. During the preparation of the final version of the manuscript, the bottom row of photos in Figure 7B were mistakenly not removed. These images may be misleading because they are only for one hydrogel (HEa-EtOH). The deleted images are not discussed in the main text and do not add anything new to the manuscript. The corrected Figure 7 and its caption appear below.

**FIGURE 7:**
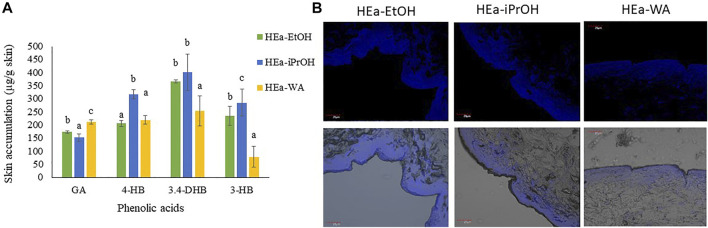
Accumulation in the skin of phenolic acids after 24-penetration; different letters indicate significant differences between the HEas, *α* = 0.05 **(A)**. The microscopic photos of vertical slicing of porcine pig skin sections 24 h after applying the HEas. The polyphenols are visible in the upper layer of the skin, along the SC. Visible are the polyphenols blue under a fluorescence effect (blue color) **(B)**. HEa-EtOH—hydrogel containing dry ethanolic extract of *E. angustifolium*; HEa-iPrOH—hydrogel containing dry isopropanol extract of *E. angustifolium*, HEa-WA-hydrogel containing dry water extract of *E. angustifolium*.

Furthermore, there was an error in **Supplementary Material**. The captions are missing for the Supplementary Videos. The correct captions appear below.

The authors apologize for this error and state that this does not change the scientific conclusions of the article in any way. The original article has been updated.

## Supplementary material

Supplementary Video S1 CTRL.

Supplementary Video S2 HEa-iPrOH.

Supplementary Video S3 HEa-EtOH.

Supplementary Video S4 HEa-WA.

