# Supplementary material for: Assessment of the Anti-Inflammatory, Antibacterial and Anti-Aging Properties and Possible Use on the Skin of Hydrogels Containing Epilobium angustifolium L. Extracts
Source: Front Pharmacol. 2022 Jul 1;13:896706. doi: 10.3389/fphar.2022.896706 (PMC9284006; doi:10.3389/fphar.2022.896706)
Supplement: Supplementary file 4 [file DataSheet1.pdf]

Assessment of the Anti-inflammatory, Antibacterial and Anti-aging Properties and Possible Use on the Skin of Hydrogels containing *Epilobium angustifolium* L. extracts

Anna Nowak [1*],· Martyna Zagórska-Dziok [2] · Magdalena Perużyńska [3], Krystyna Cybulska [4] · Edyta Kucharska [5] · Paula Ossowicz-Rupniewska [5] · Katarzyna Piotrowska [6] · Wiktoria Duchnik [7] · Łukasz Kucharski [1] · Tadeusz Sulikowski [8] . Marek Droździk [3] Adam Klimowicz [1]

[1]Department of Cosmetic and Pharmaceutical Chemistry, Pomeranian Medical University in Szczecin, Powstańców Wielkopolskich Ave Szczecin, Poland

[2]Department of Technology of Cosmetic and Pharmaceutical Products, Medical College, University of Information Technology and Management in Rzeszow, Sucharskiego 2, PL-35225 Rzeszów, Poland

[3]Department of Experimental and Clinical Pharmacology, Pomeranian Medical University in Szczecin, Powstańców Wielkopolskich Ave. 72, PL-70111 Szczecin, Poland

[4]Department of Microbiology and Environmental Chemistry, Faculty of Environmental Management and Agriculture, West Pomeranian University of Technology, 17 Juliusza Słowackiego Str., PL-71434 Szczecin, Poland

[5]Department of Chemical Organic Technology and Polymeric Materials, Faculty of Chemical Technology and Engineering, West Pomeranian University of Technology, 10 Pulaski Str. PL-70322, Szczecin, Poland

[6]Department of Physiology, Pomeranian Medical University in Szczecin, Powstancow Wlkp.72, PL-70111 Szczecin, Poland

[7]Department of Pharmaceutical Chemistry, Pomeranian Medical University, Polskiego Czerwonego Krzyża 1 Sq, PL-71244 Szczecin, Poland

[8]Clinic of General Surgery, Minimally Invasive and Gastrointestinal, Pomeranian Medical University in Szczecin,1 Uni Lubelskiej Str., PL-70111 Szczecin

1 Primary fibroblasts isolation, culture and immunofluorescence staining

Human skin samples were obtained from a volunteer, upon the approval of the Local Ethics Committee of Pomeranian Medical University (KB-0012/02/18), according to the Nejaddehbashi et al. protocol (Nejaddehbashi et al., 2019). The skin specimens were transported to the laboratory in the ice-cold Ca^{2+}/Mg^{2+}-free PBS containing 1:100 (dilution recommended for cell isolation procedures) penicillin (10,000 Units/mL)/streptomycin (10 mg/mL)/amphotericin B (25 µg/mL) solution (Sigma-Aldrich Merck Group, USA) and then processed immediately. The samples (about 1 x 1 cm^2) were then sterilized in a series of fresh 70% ethanol and rinsed with sterile PBS. The blood vessels, hypodermal adipose and epidermis were manually removed from each tissue sample, and the dermis was cut into smaller pieces. Afterwards, each piece of the tissue was placed in one drop of medium and explanted into a cell culture flask, and cultured at 37°C in 5% CO_2 in a humidified atmosphere. The medium contained DMEM high glucose (Sigma-Aldrich Merck Group, USA) supplemented with L-glutamine (2 mM; Sigma-Aldrich Merck Group, USA), antibiotic/antimycotic solution (as above) and 20% heat-inactivated fetal bovine serum. Consequently, cells were daily observed in order to control growth, and the medium (with reduced 10% FBS and 1:1000 antibiotic/antimycotic solution) was systematically changed every 3-4 days. When the cultures became confluent, the cells were serially cultured into new cell culture dishes. Afterwards, cells were routinely tested for the presence of mycoplasma contamination and frozen. Before starting the experiment, cells derived from 3 patients were pulled and immunostained for TE-7 as a fibroblast specific marker (Bliss et al., 2012). The cells were seeded at a density of 3×10^4 cells/coverslips (20 mm ⌀) and cultured as above for 48 h. In the next step, the cells were fixed in 4% buffered formalin for 5 min at 37°C, washed twice with PBS and permeabilized with 0.1% Triton X-100 in PBS for 10 min at room temperature (RT). Nonspecific antibody binding was blocked [5% normal goat serum, Sigma; for 1 h (RT)], and the cells were subsequently incubated with mouse anti-human thymic antibodies (TE7, Santa Cruz Biotechnology, USA), diluted 1:50 in blocking buffer for overnight (4°C). The coverslips were then washed thrice with 0.05% Triton X-100 in PBS. The cells were incubated with secondary anti-mouse IgG-FITC antibodies (Cat# F2012, Sigma-Aldrich Merck Group, USA) diluted 1:64 for 1 h at RT and then washed three times in PBS mounted with a mounting medium (Dako, USA) on glass slides. The slides were examined with a confocal microscope (FV1000, Olympus, Germany).

1.1 Figures

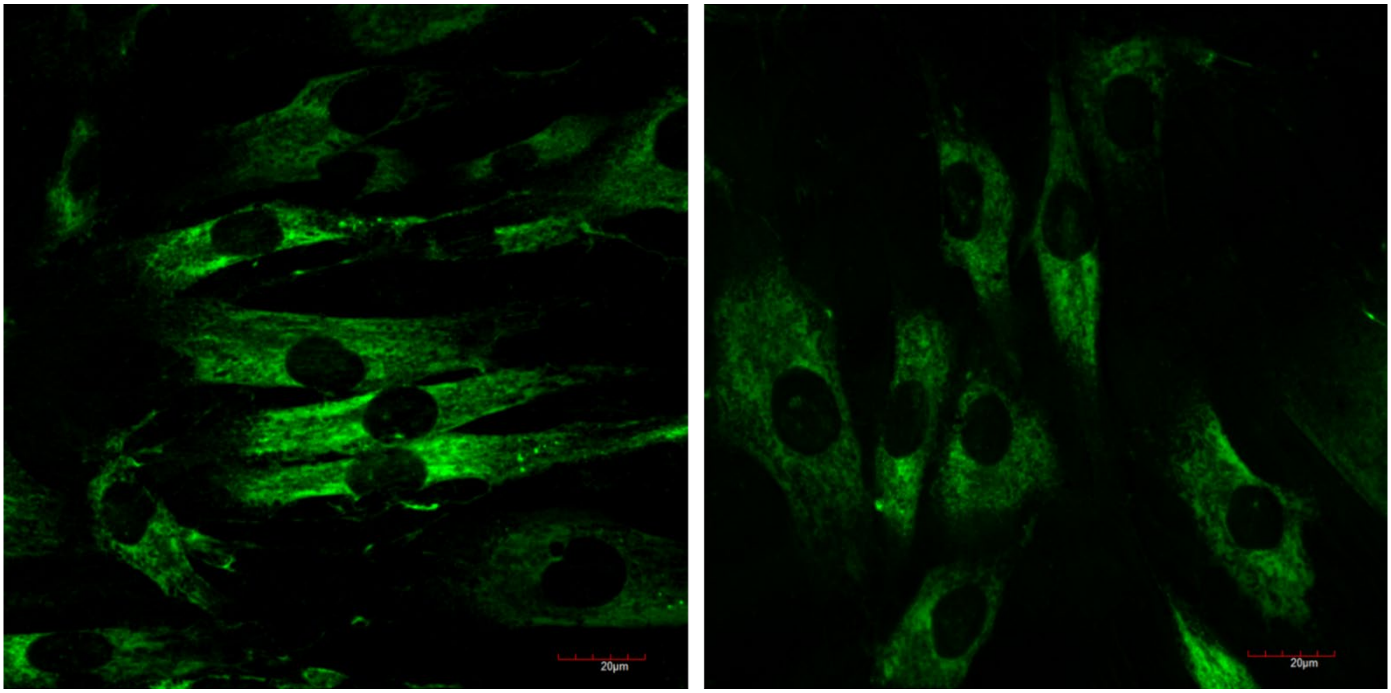

Figure S1. Detection of fibroblast specific marker (TE7) in the cytoplasm of pulled fibroblasts by the immunofluorescence staining.

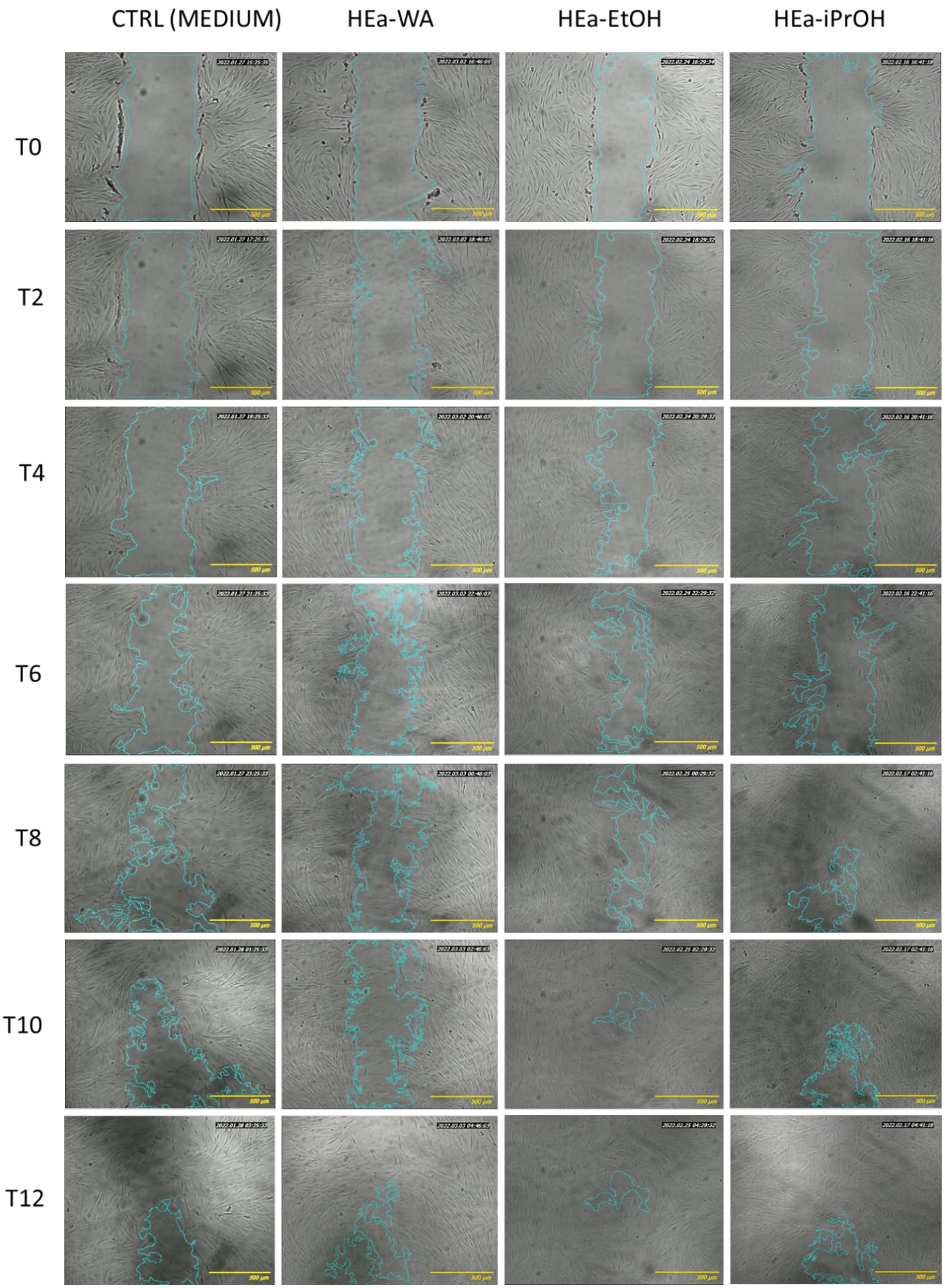

Figure S2. Representative optical microscopy images of primary human dermal fibroblast treated with hydrogels at 0.2% (100 µg/ml of *E. angustifolium* extracts) during the wound healing assay.

References

Nejaddehbashi, F., Bayati, V., Mashali, L., Hashemitabar, M., Abbaspour, M., Moghimipour, E., et al. (2019). Isolating human dermal fibroblasts using serial explant culture. *Stem Cell Investig* 6, 23–23. doi:10.21037/sci.2019.08.05.
